# Lastingly Colored Polylactide Synthesized by Dye-Initiated Polymerization

**DOI:** 10.3390/polym12091980

**Published:** 2020-08-31

**Authors:** Dawid Jędrzkiewicz, Sebastian Kowalczyk, Andrzej Plichta, Jolanta Ejfler

**Affiliations:** 1 Faculty of Chemistry, University of Wroclaw, 14 Joliot-Curie Str., 50-383 Wrocław, Poland; dawid.jedrzkiewicz@chem.uni.wroc.pl; 2 Faculty of Chemistry, Warsaw University of Technology, 3 Noakowskiego Str., 00-664 Warsaw, Poland; skowalczyk@ch.pw.edu.pl (S.K.); andrzej.plichta@pw.edu.pl (A.P.)

**Keywords:** polylactide, ring-opening polymerization, zinc, magnesium, polymer–dye conjugate

## Abstract

An efficient synthesis strategy of a well-defined polylactide–dye conjugate in a controlled fashion is presented. The introduction of coloring species as end groups of polylactide (PLA) has been performed by using new homoleptic aminophenolate magnesium or zinc coordination compounds. The molecular structure of metal complexes has been determined in solution by NMR spectroscopy, and in the solid state by X-ray analysis. Lastingly colored polymers were obtained with 2-[4-(Nitrophenylazo)-*N*-ethylphenylamino]ethanol (Disperse Red 1) and 2-[4-(2-Chloro-4-nitrophenylazo)-*N*-ethylphenylamino]ethanol (Disperse Red 13) at very high lactide conversions, based on MALDI-ToF measurement, and the macromolecules were nearly fully chain end dye-functionalized. Based on ^1^H NMR, the DP_n_ of conjugates was in the range of 10–300, which was consistent with the reaction setup. Various methods of gel-permeation chromatography (GPC) analysis were applied, and they demonstrated that the number-average molar mass (*M*_n_) values (polystyrene (PS) standards) were a bit higher than calculated, the molar mass distribution index (Ɖ_M_) values were moderate to high, the TDA (triple detection array) system was inappropriate for analysis, measurements with PDA (photo diode array) detection at 470 nm gave nearly the same molar mass distributions such as the refractometer, and the relative absorbance of conjugates at 470 nm increased linearly versus (DP_n_)^−1^. The presented approach connects the gap between the current strategy of obtaining colored polymer fibers and the design of tailor-made initiators with eco polyesters designed for the targeted applications.

## 1. Introduction

Biodegradable polymers are considered as a green alternative to petropolymers and a long-term solution for the environmentally damaging factor of plastics pollution [1,2,3]. In this class of commercially valuable polymers, polyesters are a prominent group. This set contains polylactide (PLA), which is currently the most important one on the market, because of the ecological profile of the industrial production, and also, it presents with a wide spectrum of various applications [4,5]. PLA is often referred to as *double green* because, apart from biodegradability, it is obtained from renewable raw materials. Among others, the most developing sectors are technologically advanced bioapplications of PLA for medicine and pharmacy [6,7,8,9,10,11,12]. However, it is necessary and at the same time still insufficient to search for new technologies for the production of environmentally friendly short-time commercial products for a sustainable future. Aromatic polyesters, mainly polyethylene terephthalate (PET) is dominant in the apparel industry and packaging applications; however, they are not readily degradable or recyclable [13]. In contrast, the not yet so popular PLA meets all the requirements for green polymers in terms of sustainability and degradation, which constitutes the key factors in the full cycle of eco-profile assessment of polymers using the LCA (Life Cycle Assessment) tool [4]. PLA is a material with a fairly high tensile strength; however, it is characterized by a quite high Young’s modulus (little ability to deform and low impact strength). These, among other disadvantages, are often compensated by the application of various types of chemical and physical modifications [14,15]. Chemical methods include the use of epoxy, anhydride, isocyanate, and carbodiimide (chain extenders) during processing [16], or the formation of various types of statistical, block, or grafted copolymers at the synthesis stage [17,18]. In industrial practice, physical modification methods are much more popular by introducing various types of additives that lower the price (isotropic fillers), improve strength (anisotropic fillers), increase flexibility (plasticizers), or adjust the color of compounds (dyes and pigments) [19,20]. One of the examples of a targeted application is the use of PLA in fabrics for apparel or filaments for 3D printing. PLA fiber is beneficial to the environment and is well described in the literature [21]. On the contrary, the classical process of coloration of PLA is not so green, and it requires improvement. The disperse dyeing of hydrophobic polymers such as PLA is performed in the presence of an appropriate dispersant, but that process is also not so ecological [21]. Dispersant-free dyeing with temporarily solubilized disperse dyes could extend the environmental friendliness. As a consequence, the proposed processes do not ensure color fastness or resistance to changing conditions during use, and they are prone to the uncontrolled release of dyes into the environment [21].

**Scheme 1:**
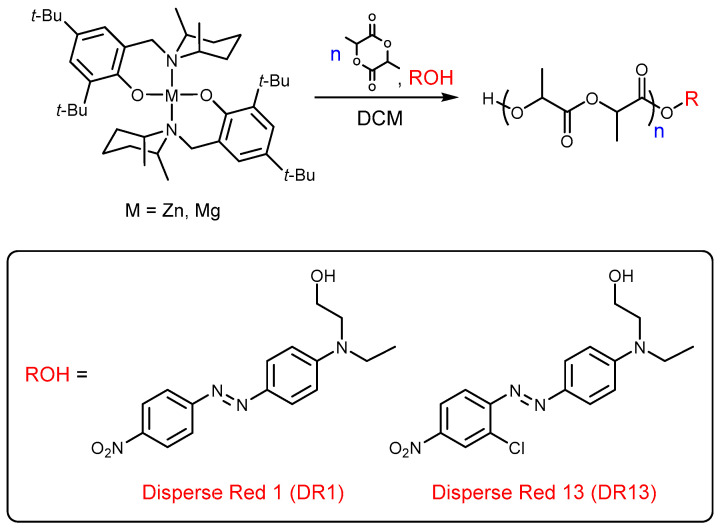
General scheme for “dye-initiated polymerization” of *L*-LA (lactide) in the presence of magnesium/zinc complexes.

The alternative greener conception could be the ring-opening polymerization (ROP) of lactide (LA) by the “dye-initiated polymerization” method, which would be used for the synthesis of colored PLA, with tailor made properties. That method allows for the synthesis of well-defined PLA–Dye conjugates, which consist of PLA chains with precisely planned lengths and with dye end groups that were obtained in a controlled fashion. In products that were obtained in this manner, the dyeing would be much longer lasting than in classic physical dyeing systems, because there will be no adverse phenomenon of migration of dyes [22,23]. The most important issue in the synthesis of PLA–Dye or other conjugates is the selection of a suitable catalyst. The industrial standard for the production of PLA is Sn(Oct)_2_, which operates for lactide polymerization in bulk conditions at elevated temperatures (110–180 °C) and, which is worth underlining, causes the number of transesterification reaction acts, so the distribution of molar masses is broad. Similarly, it is the most popular choice in conventional studies involving the ROP of cyclic esters. The versatility of this catalytic system is obvious; however, it induces also disadvantages and needs some improvements. The problem is particularly visible for the synthesis of polymers with low or ultra-low molecular weight and for those with functional or bulky end groups. Therefore, the synthesis of PLAs with well-defined properties matched to specific applications in the presence of commercial Sn(Oct)_2_ is difficult or, in some cases, impossible. Although Sn(Oct)_2_ is approved by the FDA as a food additive, the tin content in polymers should be lower than 20 ppm, as it has been found to have some cytotoxicity [24,25,26,27]. For example, the soluble tin compounds such as Sn(Oct)_2_ can be harmful at the nutrition level even at the concentration of 0.1%. In this context, the polymer obtained in the presence of tin catalyst should be handled with great care in biomedical applications. It is connected with the fact that the bulk polymerization promoted by Sn(Oct)_2_ implies that the catalyst residue remains inside the polymer material. On the other hand, the Scientific Panel on Contaminants in the Food Chain (at European Food Safety Authority) established a group-tolerable daily intake of 0.25 μg per kg of body weight for tri- and di-alkyl tin compounds [28]. This is an issue from a toxicity point of view, and the catalysts residue can also dramatically alter the polymer properties during thermal treatments at higher temperatures, thermo-modeling, or extrusion, for example [29,30,31,32]. Such rigorous requirements concerning PLA, especially for biomedical applications, are the reason for the constant search for new biocompatible lactide ROP catalysts. Therefore, even if the results obtained with Sn(Oct)_2_ are to be valued, new catalysts operating in solution under mild conditions are highly desirable to develop the further use of functional polyesters. In this aspect, the rational alternative to commercial catalysts is the catalytic systems based on biometal coordination compounds that are suitable to a given targeted application. In this context, group 1 and 2 metals, as well as zinc, are still promising candidates, and here, the most perspective and effective are single-site initiators. The synthesis of PLA–Dye conjugates is possible by using alternative appropriately designed ROP initiators, which are based on the coordination compounds of metals with a single-site motif L-M-OR (L: ancillary ligands, OR: initiating group) [33,34,35]. In such a system, dye molecules could be the fragment of the ancillary ligands or the initiating group. Moreover, this approach requires the synthesis of a new initiator for every used dye molecule. The proposed better solution here is based on the use of bifunctional catalytic systems with homoleptic coordination metal compounds, which contain an external initiating group. Catalytic systems containing homoleptic simple aminophenolate metal compounds are selectively able to produce linear alkylesters or polyesters from extra-low to high molecular weight polymer systems [36,37,38,39,40]. For this purpose, we studied our new binary catalysts composed of the (L^dmp^)_2_M compounds (L^dmp^-2,6-dimethylpiperidine (dmp), aminophenolate ancillary ligands, M–Zn, Mg), and the dye molecule possessing the hydroxyl functionalization DR1/13 (Scheme 1). 

Herein, we report a new catalytic system containing precisely design ancillary ligands that prevent the metal center against aggregation, and additionally, ligands redistribution reactions in the presence of the bulky external initiating group. The homoleptic structural motifs of magnesium and zinc complexes have been confirmed by X-ray diffraction studies. Detailed analysis of the presence of corresponding species in solution indicated their dynamic behavior induced by the de-coordinating of amine arm of ancillary ligands. That “gorilla effect” regarding dynamism plays a crucial role in the polymerization reaction. The experimental data should allow for a new insight on the design of effective catalytic systems that ensure the fit of both an ancillary and initiating group—for example, dye molecule during the ROP reaction. The presented catalysts work in mild conditions and produce PLA with both short and longer polymers chains in a few minutes. The proposed so-called “dye-initiated polymerization” method gives the possibility for the synthesis of colored PLA with stable/long-lasting and planned saturation of PLA fabric color, which is controlled by a polymer chain length.

## 2. Materials and Methods 

The synthesis of complexes and polymerization reactions, which required an inert atmosphere of N_2_, was performed by using a glove-box (MBraun) or standard Schlenk apparatus and vacuum line techniques.

Solvents for synthesis were purified by standard methods: MeOH (HPLC, VWR) distilled over Mg, dichloromethane (99.8% VWR), and *n*-hexane (HPLC, VWR) was dried and purified using the Solvent Purification Systems (Inert, PureSolv EN 1-7 Base), C_6_D_6_ was distilled over CaH_2_. *L*-LA ((3*S*)-cis-3,6-dimethyl-1,4-dioxane-2,5-dione) (98%; Aldrich) was recrystallized from toluene and sublimed prior to its use. Chemicals that were obtained from commercial sources were used without further purification: 2,4-di-*tert*-buthylphenol (99%, Sigma-Aldrich, St. Louis, MO, USA), *cis*-2,6-dimethylpiperidine (97%, Sigma-Aldrich), formaldehyde (37% solution in H_2_O, Sigma-Aldrich), diethylzinc solution (1.0 M in heptane, Sigma-Aldrich), di-*n*-butyl-magnesium solution (1.0 M in heptane, Sigma-Aldrich), Disperse Red 1 (2-[4-(Nitrophenylazo)-*N*-ethylphenylamino]ethanol, dye content 95%, Sigma-Aldrich), Disperse Red 13 (2-[4-(2-Chloro-4-nitrophenylazo)-*N*-ethylphenylamino]ethanol, dye content 95%, Sigma-Aldrich).

The NMR spectra were recorded at 298 K using a Bruker Avance 500 MHz spectrometer. Chemical shifts are reported in ppm and referenced to the residual protons in the deuterated solvent (C_6_D_6_, ^1^H: 7.16 ppm, ^13^C: 128.06 ppm) [41]. HRMS spectra were recorded using Bruker MicOTOF-Q spectrometers with an Electrospray ionization technique(ESI) and time-of-flight mass analyzer. Microanalyses were conducted with an Elementar CHNS Vario EL III analyzer. The number-average molar mass (*M_n_*) and the molar mass distribution index (Ɖ_M_) of the samples were determined by gel-permeation chromatography (GPC). The system was composed of a Viscotek GPCmax unit (pumping and degassing of solvent, sample injection with autosampler), a 305 TDA detection unit (consisting of column, a UV measuring cell, RI detector, hybrid Right-Angle Light Scattering/ Low-Angle Light Scattering (RALS/LALS) detectors, and a viscometer), and a PDA UV detector (190–500 nm). The system was equipped with a Jordi Labs DVB column (mixed bed, 5 μm), which worked with dichloromethane at 30 °C, with a flow rate of 1 cm^3^/min. The polymer populations (including repeating units and end groups) were characterized by a MALDI-ToF system. The spectrometer used was Bruker ultrafleXtreme, measurements were carried out in linear mode, with a DCTB (2-[(2E)-3-(4-tert-Butylphenyl)-2-methylprop-2-enylidene]malononitrile) matrix and potassium as an ion source.

X-ray diffraction data for a suitable crystal of each sample were collected using an Xcalibur CCD Ruby with a ω scan technique. The data collection and processing utilized the CrysAlis suite of programs [42]. The space groups were determined based on systematic absences and intensity statistics. Lorentz polarization corrections were applied. The structures were solved using intrinsic phasing SHELXT-2014/5 and refined by full-matrix least-squares on F^2^. All calculations were performed using the SHELX suite of programs [43]. All non-hydrogen atoms were refined with anisotropic displacement parameters. Hydrogen atom positions were calculated with geometry and were not allowed to vary. Thermal ellipsoid plots were prepared with 50% of probability displacements for non-hydrogen atoms by using the Mercury 3.9 program [44]. All of the data have been deposited with the Cambridge Crystallographic Data Centre CCDC-1982025 for (L^dmp^)_2_Zn and -1982026 for (L^dmp^)_2_Mg. Copies of the data can be obtained free of charge by application to CCDC, 12 Union Road, Cambridge CB21EZ, UK or e-mail: deposit@ccdc.cam.ac.uk.

Syntheses details are presented in Appendix A.

## 3. Results and Discussion

**Scheme 2:**
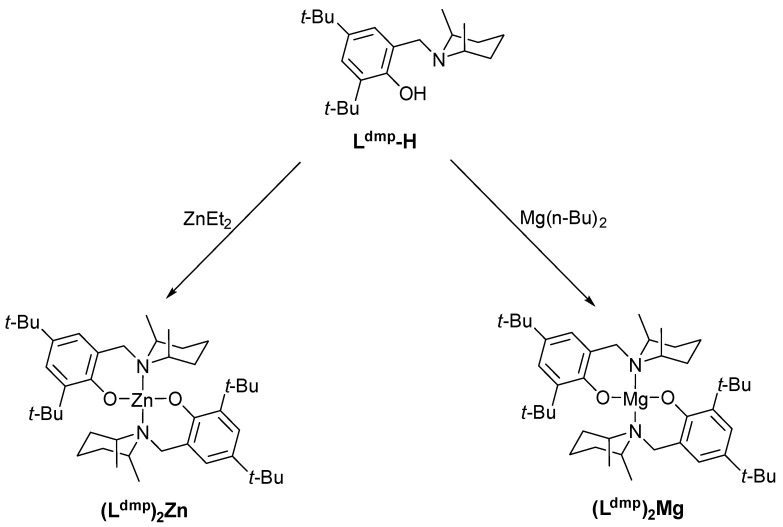
Synthesis of zinc and magnesium aminophenolates.

The basic components of catalytic systems for PLA–Dye conjugates include the homoleptic aminophenolate metal complexes (L^dmp^)_2_M (M = Mg, Zn) and dye molecules with a hydroxyl terminal group. In our findings, the ancillary ligand L^dmp^-H has been obtained by a standard Mannich condensation reaction between 2,4-di-*tert*-buthylphenol and *cis*-2,6-dimethylpiperidine. Next, it was used for the new homoleptic zinc and magnesium compounds syntheses by a clean reaction with commercially available metal precursors MR_2_ (e.g., Mg(*n*-Bu)_2_ or ZnEt_2_). The reaction was carried out readily at room temperature in *n*-hexane, because aminophenolate ligands with a sizable substitution in the ortho positions of the aryl core afforded expected bis-chelation products without stoichiometry control (Scheme 2) [38]. 

**Figure 1:**
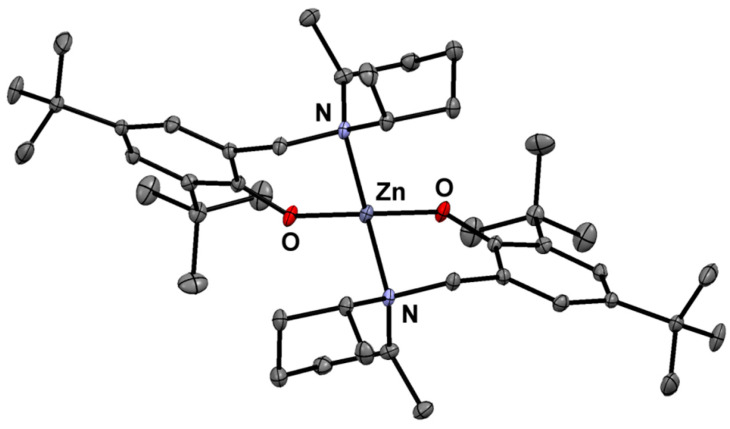
Molecular structure of (L^dmp^)_2_Zn.

**Figure 2:**
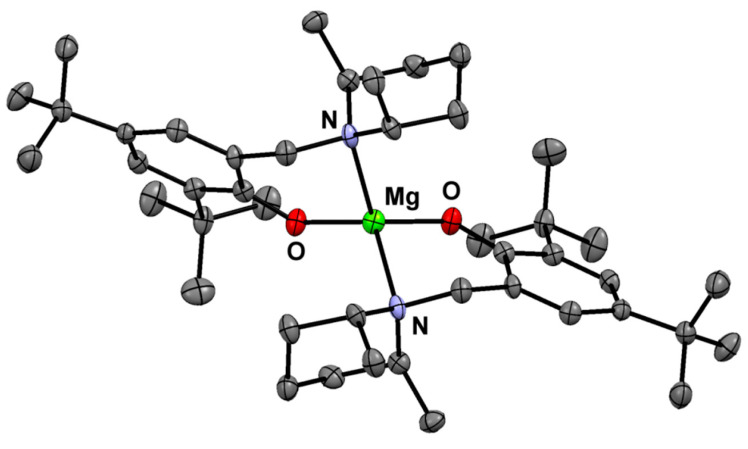
Molecular structure of (L^dmp^)_2_Mg.

The aminophenol and metal compounds were obtained in high yields—L^dmp^-H (85%), (L^dmp^)_2_Zn (87%), and (L^dmp^)_2_Mg (83%), respectively—and characterized by standard elemental analysis and NMR spectroscopy (for details see, the Experimental Section and Supplementary Materials–Figures S1–S16. The molecular structures of bis-chelate metal compounds were determined by X-ray analysis. The solid-state structures of obtained zinc and magnesium compounds are presented in Figure 1 and Figure 2, respectively, and Table S1 with summarized crystal data. Both zinc and magnesium compounds are isostructural and reveal the expected monomeric nature. The metal centers in the magnesium and zinc compounds adopt distorted tetrahedral geometries with typical bond lengths for Zn–O, Mg–O, Zn–N, and Mg–N that are similar in characteristic ranges for the related compounds described previously [39,40,45,46,47,48,49,50] (see ESI, Table S3). 

**Figure 3:**
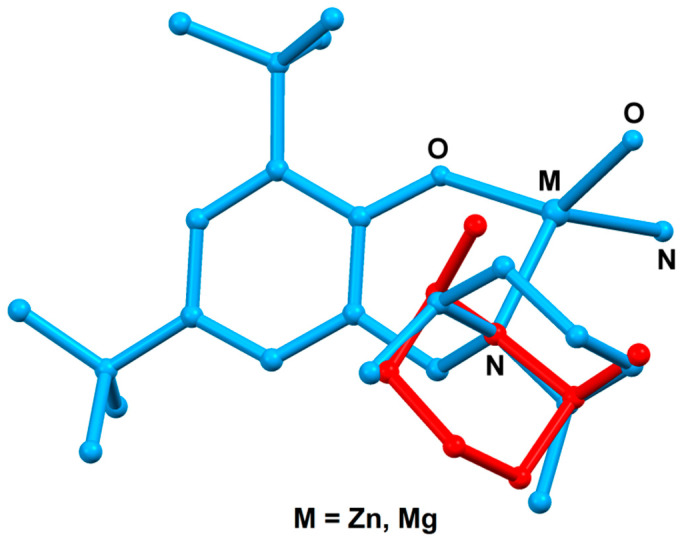
Disordered dimethylpiperidinyl groups observed in molecular structures of zinc and magnesium complexes.

In both molecular structures, the dimethylpiperidinyl group of the ligand is disordered in two positions with occupation factors of 0.929(4) (blue) and 0.071(4) (red) for Zn, and 0.873(5) (blue) and 0.127(5) (red) for Mg, respectively (Figure 3).

**Figure 4:**
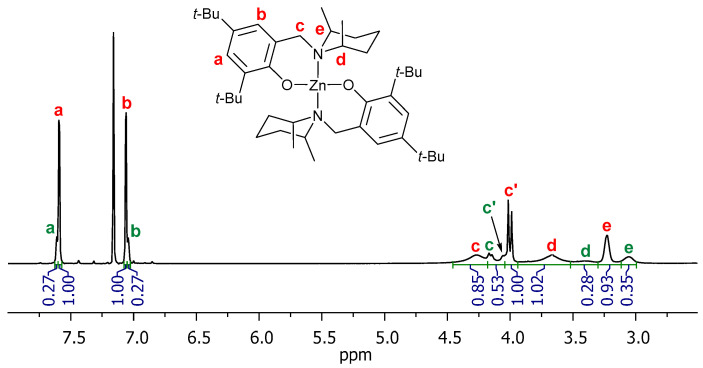
Fragment of ^1^H NMR spectrum of (L^dmp^)_2_Zn (C_6_D_6_). Major form: red, minor form: green.

**Figure 5:**
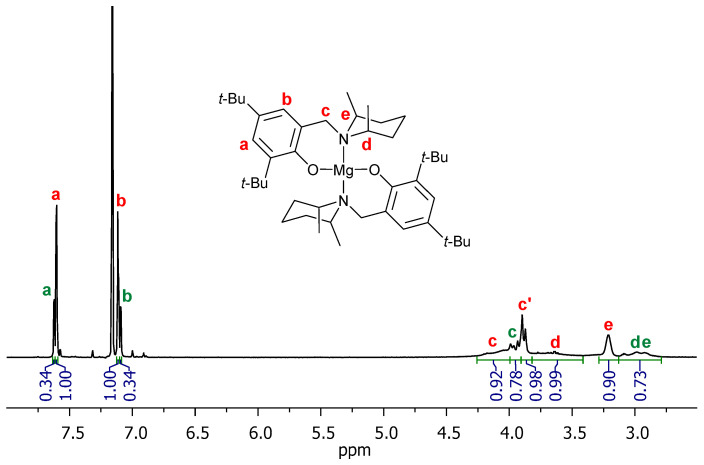
Fragment of ^1^H NMR spectrum of (L^dmp^)_2_Mg (C_6_D_6_). Major form: red, minor form: green.

Although the molecular structures of the magnesium and zinc bis-chelates are clear and anticipated in the solid state, in solution, a mixture of isomers is formed (Figure 4 and Figure 5). Such a phenomenon is expected for homoleptic aminophenolates, and this fact results in the prochiral auxiliary ligands located around the metal center, which after their coordination, induce dynamic behavior in the solution [39]. The most significant difference between potential isomers in solution is a mutual position of substituents at the nitrogen atoms and their transformation by decoordination of the amine arm of the ligand. The ^1^H NMR spectra of (L^dmp^)_2_M (M = Zn, Mg) contain two sets of signals (ratio 1:0.27 for Zn, 1:0.34 for Mg), indicative toward two potential isomers in the solution, which most likely correspond to a dangling effect of the amine arm or a disorder observed in the solid state. The pattern of the diastereotopic methylene signals suggests the “gorilla” effect (quick coordination and decoordination of amine arm interchangeably), which was discussed earlier for similar aminophenolate bis-chelates [39]. The dynamic behavior in the solution, induced by such bond-dangling, changes the general geometry around the metal center, and in turn improves the catalytic activity of bis-chelate complexes [40].

**Figure 6:**
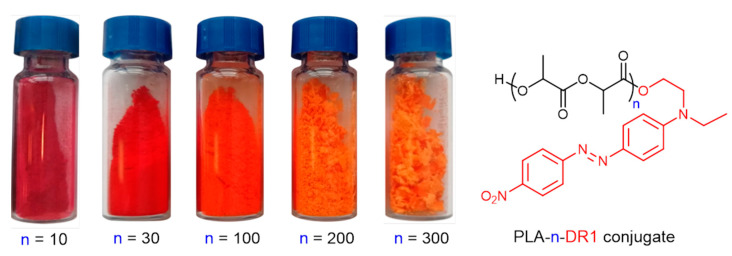
Polylactide (PLA)-n-DR1 conjugate samples with controlled molecular weights.

**Figure 7:**
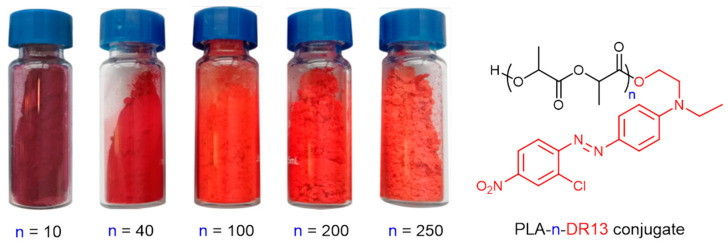
PLA-n-DR13 conjugate samples with controlled molecular weights.

The next step was the verification of the activity of the catalytic system based on structurally analogous bis-chelates L_2_M and dye molecules as external initiating groups in the ROP of *L*-LA. The proposed so-called “dye-initiated polymerization” method gives the possibility for the synthesis of colored PLA with a stable and planned saturation of PLA fabric color, which is controlled by a polymer chain length (Figure 6 and Figure 7).

**Figure 8:**
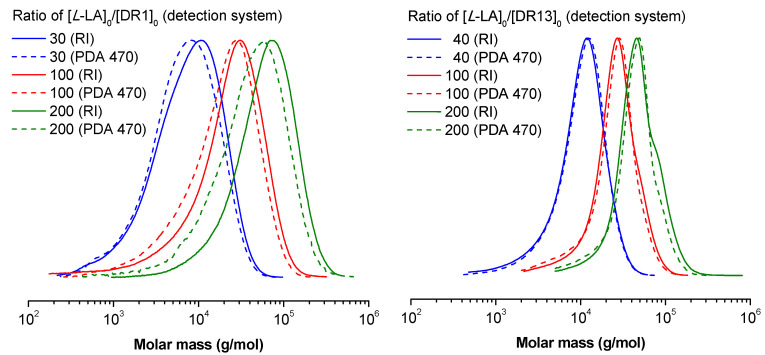
Gel-permeation chromatography (GPC) traces for selected PLA-n-DR obtained from various detection systems: refractometer (solid lines) and photo diode array (PDA) detector at 470 nm (dashed lines) for various ratios of [*L*-LA]_0_/[ROH]_0_ for polymerization initiated with DR1 (left plot) or DR13 (right plot) series.

The study of ROP polymerization of the tested compounds has been investigated under comparable reaction conditions by using the same dye molecules (Disperse Red 1 –DR1, Disperse Red 13–DR13) with different molar ratios of (L^dmp^)_2_M/*L*-LA/DR = 1/n/1. The ROP process was monitored by using NMR spectroscopy; all polymerization reactions, which were carried out at room temperature, achieved high conversion rates (>95%) in several minutes. The polymerization results obtained for (L^dmp^)_2_M/DR systems are summarized in Table 1. The homoleptic bis-chelate magnesium and zinc compounds show decent control over the average molar mass of polymers obtained in the ROP processes. The obtained conjugates of PLA-n-DR (where, n is the number of LA monomeric units, DR1 or DR13 indicates the dye molecule applied for initiation) showed moderate to large values of Ɖ_M,PS_ while calculated with conventional calibration based on PS standards. The M_n,PS_ values calculated with the same method were similar to the ones calculated (M_n,cal_) with respect to the fully controlled process with a known initial monomer to initiator ratio ([L-LA]_0_/[ROH]_0_) and monomer conversion (p*_L_*_-LA_) in case of low DP_n_ values in the range of 10–40 (nos. 1, 2, 6, 7). On the other hand, for samples with DP_n_ of 100 or higher (nos. 3–5, 10, 11) the M_n, PS_ tends to significantly exceed the calculated values. For some unknown reason, the two samples nos. 8 and 9 with a DP_n_ value of 100 initiated with DR1 showed lower M_n,PS_ values than M_n,cal_. It is probably caused by more intensive transesterification processes, which may be proved by broad molar mass distribution and higher Ɖ_M_ values. The rules presented above are unrelated to kind of metal atom present in the catalyst structure. Additionally, gel-permeation chromatography (GPC) results were calculated with an absolute calibration method, using a triple detection array (TDA) system. Ɖ_M,TDA_ values were much lower than the Ɖ_M,PS_ ones; however, there was no reasonable correlation found between M_n,TDA_ results and the other M_n_ values, although some individual TDA results were very similar to those calculated. In this particular case, the TDA results might not be valuable, especially for polymers of low DP_n_ and broad distribution, because of the huge differences in the refractive index increments for PLA and DR. Therefore, the DR end groups were much better “seen” by the Refractive Index (RI) detector, which deflects the real concentration particularly of shorter chains, bending self-calibration curve and hence the results. Summarizing, one can say that better control in terms of the ƉM might be achieved when DR13 was used as a co-initiator than in the case of DR1, where the metal atom present in the initiator molecule had no effect. The comparison of molar mass distribution measured with an RI detector and PDA detector at 470 nm (DR molecules absorb at that wavelength, whereas PLA monomeric units do not) showed that in the majority of samples, the distribution of dye overlaps fairly well with the distribution of the polymer (Figure 8), as well as with the monomodal distributions in general. Furthermore, the GPC traces and experimental Mn estimated within both detection systems are shifted toward higher molar masses, once the ratio of [*L*-LA]_0_/[ROH]_0_ increased.

**Figure 9:**
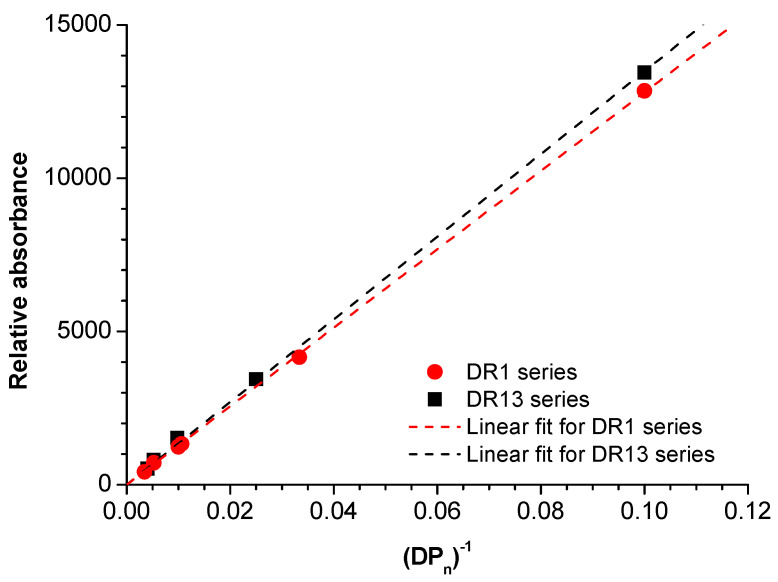
Relative absorbance of the PLA-n-DR at 470 nm versus inverse DP_n_ for DR1 (red circles) and DR13 (black squares) series; points are experimental data, whereas dashed lines are linear fits for that data.

Then, a plot of relative absorbance at 470 nm versus the inverse DPn was also prepared for all samples broken down by the type of DR. Relative absorbance was determined by dividing the area under the molar mass distribution measured with GPC, equipped with a PDA detector at 470 nm by the sample concentration and injection volume. The experimental data of both DR series fit famously to a linear trend (determination coefficients R2 > 99%); however, the slopes of the series are slightly different due to the nature of the chromophore (Figure 9). The plot shows that a color intensity of the polymer may be precisely adjusted by the initial ratio of monomer to initiator, as well as blending with undyed PLA. Moreover, the kind of metal does not affect the trend, so from that aspect, both types of initiators are appropriate.

MALDI-ToF analysis of obtained products revealed that the population of linear macromolecules initiated with respective DR, and the series of macrocycles of even and odd numbers of lactic acid monomeric units were present (Table 2, Table S1, Figures S17–S24). The linear products, which formally initiated with water molecules, were discovered only in the case of DR13 series with low DP_n_ (Table 2, no. 1 and 2); however, a fraction of a number of molecules in that population was merely 2%. It proves that the ROP was exclusively initiated with DR alcohols. The presence of chains comprising odd numbers of lactic acid repeating units, as well as macrocyclic products in all samples, indicate that transesterification processes took place in the system, which broadened distributions of molar masses. The fraction of number of molecules in a population for macrocyclic products increases for a higher ratio of [*L*-LA]_0_/[ROH]_0_. However, those data are strongly distorted by the poor ability of long linear chains present in those samples to undergo flight in the MALDI system, comparing with the macrocyclic population which is rather of similar and quite low Mn in all samples (Table S1). Therefore, we think that the most accurate results among the set of experiments presented in Table 2 are for samples nos. 1, 2, 6, and 7, in which 0%, 4%, 0%, and 2.4% of cyclic macromolecules have been demonstrated, respectively. The macrocyclization processes caused the odd and even numbers of lactic acid monomeric units in PLA molecules to be comparable to each other in most of the cases. The samples containing very low DP_n_, had even numbers, which were c.a. 1.5–1.7 times higher than odd ones.

**Figure 10:**
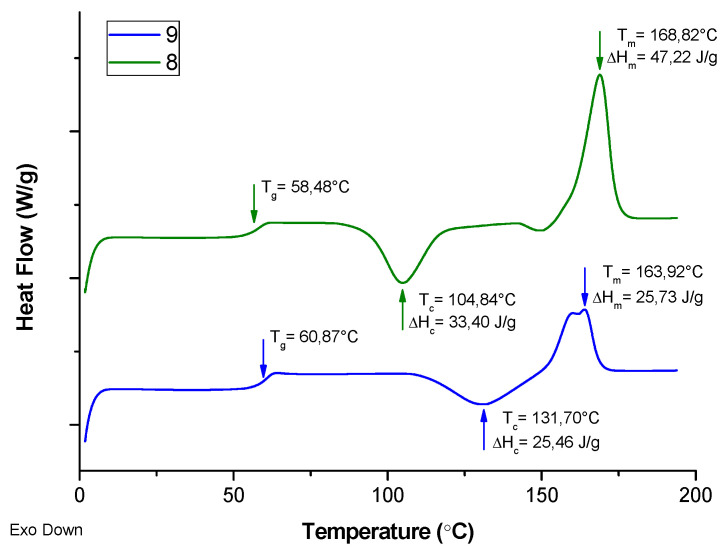
DSC curves of second heating cycle for products no. 8 and 9 (corresp. to Table 1).

Differential Scanning Calorimetry (DSC) measurements were carried out for samples nos. 8 and 9, which were obtained with the same co-initiator (DR1) and at the same targeted DP_n_ of 100, but in the presence of different catalysts comprising of Zn and Mg species, respectively (Figure 10, second heating). It showed that the values of T_g_ of these samples are similar, whereas sample no. 9 revealed that its temperature of cold crystallization is higher, and the melting temperature is lower than for sample no. 8. Similarly, the energetic effect of melting of sample no. 9 is almost as twice as low than for sample no. 8. It demonstrates that the ability to crystallize in the case of the sample obtained in the presence of Mg based catalyst (no. 9) is lower, and it might be caused by the less regular microstructure of the polymer chain. Although the samples were synthesized using L-LA, and therefore they should provide fully isotactic PLA, some racemization side reactions could occur during or after polymerization in the presence of the Mg complex, so in turn, the polymer microstructure was potentially affected and the ability for the sample to crystallize diminished. 

**Figure 11:**
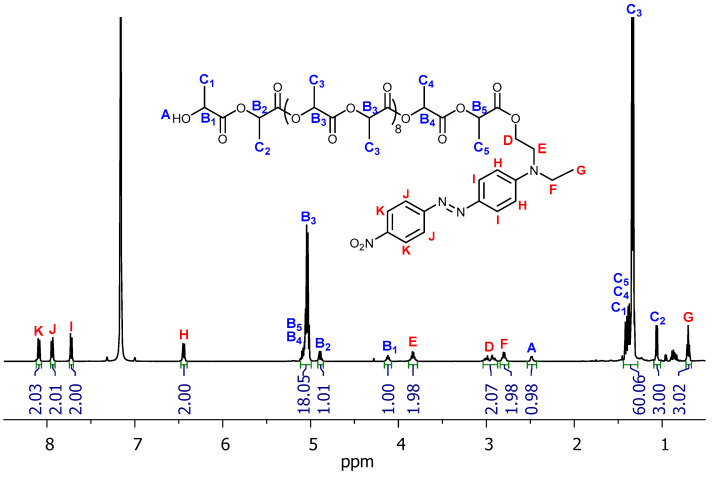
^1^H NMR spectrum of PLA-10-DR1 (C_6_D_6_).

**Figure 12:**
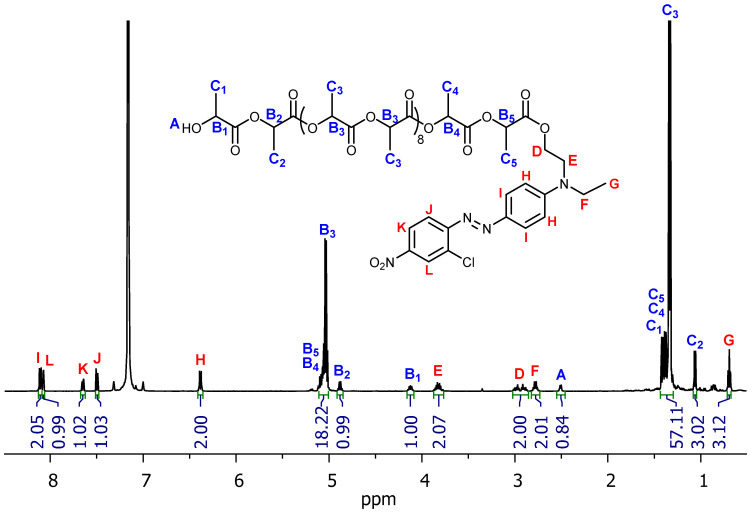
^1^H NMR spectrum of PLA-10-DR13 (C_6_D_6_).

The ^1^H NMR spectra with proton signals assigned to the structures of exemplary oligomeric conjugates PLA-10-DR1 and PLA-10-DR13 are shown in Figure 11 and Figure 12. The spectra showed the expected chain ends: resonances due to dye molecules (denoted as red letters D–K) and a hydroxyl group (blue letter A). The most intensive signals are multiplets at 5.04 ppm (blue B_3_) and 1.33 ppm (blue C_3_), corresponding to methine (CH) and methyl (CH_3_) groups, respectively. These groups are present in the oligolactide, which consists of eight repeating units. The adequate multiplets denoted as B_1_, B_2_ at 4.12, 4.89 ppm correspond to CH. Doublets marked as C_1_, C_2_ at 1.41, 1.06 ppm are for CH_3_, which refer to the unit located in the vicinity of the hydroxyl chain end. Quartets (B_4_, B_5_ at range 5.12–5.07 ppm) and doublets (C_4_, C_5_ at 1.36, 1.39 ppm) correspond to the unit located next to the dye chain end.

## 4. Conclusions

The new homoleptic magnesium and zinc complexes active in the ROP of LA have been synthesized. The molecular structures of bis-chelate metal compounds have been determined by X-ray analysis. Both zinc and magnesium compounds are isostructural with a monomeric nature, where the metal centers adopted distorted tetrahedral geometries. Although the molecular structures of the magnesium and zinc bis-chelates are clear in the solid state, in solution, a mixture of two isomers are afforded, corresponding to the dynamic coordination/de-coordination of an amine arm. Therefore, the dynamic behavior in solution, which was induced by such amine arm-dangling, changes the general geometry around the metal center, allowing for easier bonding of the LA monomer to the metal center, which in consequence improves the catalytic activity of bis-chelate complexes. Therefore, LA polymerizations catalyzed with these species and co-initiated with Disperse Red 1 and 13 molecules allowed achieving very high monomer conversion in just minutes at room temperature, and they resulted in almost fully chain end dye-functionalized PLAs of even concentration of colorant molecules within the distribution of polymer molar mass. On the other hand, the molar mass distributions are quite broad mainly due to transesterification processes; however, it does not matter significantly from the point of view of fiber application. The self-colored polymers of higher DP_n_ could be used as fibers separately, whereas the conjugates of low molar mass can be introduced to blends with commercial PLA as non-migratory and miscible/compatible colorants. Summarizing, the proposed “dye-initiated polymerization” method gives the “greener” possibility for the synthesis of lasting colored PLA with a stable and planned saturation of PLA fabric color controlled by polymer chain length.

## Figures and Tables

**Table 1:** Ring-opening polymerization (ROP) of *L*-LA initiated by zinc and magnesium complexes with Disperse Red 1 (DR1) and Disperse Red 13 (DR13) as co-initiators.

No.	Initiator [I]	ROH	Molar Ratios *^a^*	Time (min.)	*p_L_*_-LA_ (%) *^b^*	DP_n_ *^c^*	M_n,cal_ *^d^*	CC-RI-GPC*^e^*		TDA-GPC *^f^*	
								M_n,PS_	Ɖ_M,PS_	M_n,TDA_	Ɖ_M,TDA_
1	(L^dmp^)_2_Zn	DR13	1/10/1	5	100	10	1.79	1.78	1.90	6.87	1.10
2	(L^dmp^)_2_Zn	DR13	1/40/1	5	100	40	6.11	6.79	1.81	12.9	1.04
3	(L^dmp^)_2_Zn	DR13	1/100/1	5	99.1	102	14.6	19.8	1.52	14.6	1.28
4	(L^dmp^)_2_Zn	DR13	1/200/1	10	97.3	192	28.4	34.8	1.55	31.6	1.10
5	(L^dmp^)_2_Mg	DR13	1/250/1	15	99.1	248	36.1	43.7	1.51	29.8	1.31
6	(L^dmp^)_2_Zn	DR1	1/10/1	5	100	10	1,76	1.39	2.00	3.77	1.05
7	(L^dmp^)_2_Zn	DR1	1/30/1	5	100	30	4.64	4.81	2.29	9.29	1.02
8	(L^dmp^)_2_Zn	DR1	1/100/1	5	99.8	100	14.7	10.4	3.12	15.5	1.25
9	(L^dmp^)_2_Mg	DR1	1/100/1	5	97.8	94	14.4	10.3	3.74	52.5	1.50
10	(L^dmp^)_2_Mg	DR1	1/200/1	15	99.6	190	29.0	38.9	1.97	21.6	1.63
11	(L^dmp^)_2_Mg	DR1	1/300/1	30	99.9	289	43.5	55.8	2.06	36.2	1.72

Reaction conditions: V_solvent_ = 20 mL, CH_2_Cl_2_; T = 25 °C; general remarks: M_n,cal_ expressed in g/mol; M_n,PS_ and M_n,TDA_ extressed in kg/mol; remarks: *^a^* initial molar ratio of [I]_0_/[*L*-LA]_0_/[ROH]_0_; *^b^* conversion of monomer estimated by ^1^H NMR; *^c^* degree of polymerization estimated by ^1^H NMR; *^d^* calculated from the formula of M_n,cal_ = [*L*-LA]_0_/[ROH]_0_ × *p_L_*_-LA_ × 144.13 + M_ROH_; ^*e*^ determined by GPC calibrated versus polystyrene standards; *^f^* determined by GPC with TDA based on known sample concentration [51].

**Table 2:** Fraction of number of molecules in population (by MALDI-ToF) of PLA obtained by ROP of *L*-LA initiated by zinc and magnesium complexes with Disperse Red 1 (DR1) and Disperse Red 13 (DR13) as co-initiators.

No. (Corresp. to Table 1)	Fraction of Number of Molecules in Population (%) *^a^*							
	Macrocycles		H–(LA)_n_–OH		DR1–(LA)_n_–OH		DR1–(LA)_n_–OH	
	Even	Odd	Even	Odd	Even	Odd	Even	Odd
1			1.3				62.1	36.6
2	1.9	2.1	1.1	0.9			52.3	41.7
3	11.4	11.7					39.1	37.8
4	12.8	13.5					38.0	35.7
6					60.4	39.6		
7	1.2	1.2			48.7	48.9		
8	5.6	5.3			44.4	44.7		
9	9.1	9.1			41.9	39.9		
10	6.9	7.1			43.0	43.0		
11	20.2	21.2			29.6	29.0		

General remarks: empty field means ‘no population observed’; remarks: *^a^* determined by MALDI-ToF measurement: populations of PLA chains comprised of even and odd numbers of lactic acid monomeric units.

## Supplementary Materials

The following are available online at https://www.mdpi.com/2073-4360/12/9/1980/s1, Figure S1. ^1^H NMR of L^dmp^-H in C_6_D_6_. Figure S2. ^13^C NMR of L^dmp^-H in C_6_D_6_. Figure S3. ^1^H NMR of (L^dmp^)_2_Zn in C_6_D_6_. Figure S4. ^13^C NMR of (L^dmp^)_2_Zn in C_6_D_6_. Figure S5. ^1^H COSY of (L^dmp^)_2_Zn in C_6_D_6_. Figure S6. ^1^H NOESY of (L^dmp^)_2_Zn in C_6_D_6_. Figure S7. ^1^H NMR of (L^dmp^)_2_Mg in C_6_D_6_. Figure S8. ^13^C NMR of (L^dmp^)_2_Mg in C_6_D_6_. Figure S9. ^1^H COSY of (L^dmp^)_2_Mg in C_6_D_6_. Figure S10. ^1^H NOESY of (L^dmp^)_2_Mg in C_6_D_6_. Figure S11. ^1^H NMR of PLA-10-DR1 in C_6_D_6_. Figure S12. ^13^C NMR of PLA-10-DR1 in C_6_D_6_. Figure S13. ^1^H COSY of PLA-10-DR1 in C_6_D_6_. Figure S14. ^1^H NMR of PLA-10-DR13 in C_6_D_6_. Figure S15. ^13^C NMR of PLA-10-DR13 in C_6_D_6_. Figure S16. ^1^H COSY of PLA-10-DR13 in C_6_D_6_. Table S1. Results of MALDI ToF on ROP of *L*-LA initiated by zinc and magnesium complexes with Disperse Red 1 (DR1) and Disperse Red 13 (DR13) as co-initiators. Figures S17‒S24. MALDI ToF mass spectra for obtained polymers. Table S2. X-ray experimental data and refinement for (L^dmp^)_2_Zn and (L^dmp^)_2_Mg. Table S3. Selected bond distances and angles for (L^dmp^)_2_Zn and (L^dmp^)_2_Mg.

## Appendix A

### Appendix A.1. Syntheses

#### Appendix A.1.1. 2:4-di-tert-butyl-6-(((2R,6S)-2,6-dimethylpiperidin-1-yl)methyl)phenol, L^dmp^-H

To a solution of 1.50 g (7.20 mmol) of 2,4-di-*tert*-butylphenol and 1.00 mL (7.20 mmol) of *cis*-2,6-dimethylpiperidine in methanol (50 mL), 0.80 mL (10.63 mmol) of formaldehyde (37% solution in H_2_O) was added. The solution was stirred and heated under reflux for 24 h. Then, it was concentrated in vacuo and it was placed at −15 °C until a product precipitated as a white crystalline solid. It was collected by filtration, washed with cold methanol, and dried in vacuo to give L^dmp^-H. Yield 85% (2.03 g, 6.12 mmol). ^1^H NMR (500 MHz, C_6_D_6,_ RT) δ: 12.71 (br s, 1H, OH), 7.43 (d, *J*_HH_ = 2.3 Hz, 1H, ArCH), 6.85 (d, *J*_HH_ = 2.3 Hz, 1H, ArCH), 3.67 (s, 2H, Ar-CH_2_-N), 1.98 (br s, 2H, N-CH), 1.73 (s, 9H, C(CH_3_)_3_), 1.39 (s, 9H, C(CH_3_)_3_), 1.38–1.31 (m, 1H, CH_2_), 1.28–1.18 (br s, 2H, CH_2_), 1.19–1.09 (m, 2H, CH_2_), 1.08–1.01 (m, 1H, CH_2_), 0.97 (br s, 6H, CH_3_). ^13^C NMR (126 MHz, C_6_D_6_, RT) δ: 155.9 (s, 1C, ArC-OH), 140.2 (s, 1C, ArC), 136.4 (s, 1C, ArC), 123.3 (s, 1C, ArC), 121.9 (s, 1C, ArCH), 121.1 (s, 1C, ArCH), 60.0 (s, 2C, N-CH), 57.9 (s, 1C, Ar-CH_2_-N), 35.4 (s, 1C, C(CH_3_)_3_), 35.2 (s, 2C, CH_2_), 34.4 (s, 1C, C(CH_3_)_3_), 32.1 (s, 3C, C(CH_3_)_3_), 30.0 (s, 3C, C(CH_3_)_3_), 25.6 (s, 1C, CH_2_), 21.3 (s, 2C, CH_3_). HRMS(ESI): calcd for C_22_H_37_NO: 332.291 [M + H]+, found 332.29. Elemental Anal. calcd (found) for C_22_H_37_NO: C, 79.70 (78.48); H, 11.25 (11.66); N, 4.22 (4.06) %.

#### Appendix A.1.2. (L^dmp^)_2_Zn

To a solution of L^dmp^-H (0.66 g, 2.00 mmol) in *n*-hexane (20 mL), ZnEt_2_ (1 mL, 1.00 mmol) was added drop-wise at room temperature. The solution was stirred for 24 h, and then it was concentrated in vacuo and placed at −15 °C until a product was precipitated as colorless crystals. It was filtered off, washed with *n*-hexane (10 mL), and dried in vacuo to give (L^dmp^)_2_Zn. Yield 87% (0.64 g, 0.87 mmol). ^1^H NMR (500 MHz, C_6_D_6,_ RT) δ: Major form: 7.59 (d, *J*_HH_ = 2.4 Hz, 2H, ArCH), 7.06 (d, *J*_HH_ = 2.4 Hz, 1H, ArCH), 4.27 (br s, 2H, Ar-CH_2_-N), 4.00 (d, *J*_HH_ = 13.2 Hz, 2H, Ar-CH_2_-N), 3.67 (br s, 2H, N-CH), 3.23 (br s, 2H, N-CH), 1.70 (s, 18H, C(CH_3_)_3_), 1.48 (s, 18H, C(CH_3_)_3_), 1.98–1.75 (m, 4H, CH_2_), 1.63–1.53 (m, 2H, CH_2_), 1.37–1.30 (m, 4H, CH_2_), 1.26–1.15 (m, 2H, CH_2_), 1.06 (d, *J*_HH_ = 6.2 Hz, 6H, CH_3_), 0.90 (d, *J*_HH_ = 6.5 Hz, 6H, CH_3_); Minor form (selected chemical shifts): 7.61 (s, 2H, ArCH), 7.04 (s, 2H, ArCH), 4.16 (d, *J*_HH_ = 11.9 Hz, 2H, Ar-CH_2_-N), 4.04 (d, *J*_HH_ = 11.9 Hz, 2H, Ar-CH_2_-N), 3.43 (br s, 2H, N-CH), 3.06 (br s, 2H, N-CH), 1.71 (s, 18H, C(CH_3_)_3_), 1.50 (s, 18H, C(CH_3_)_3_). ^13^C NMR (126 MHz, C_6_D_6_, RT) δ: 163.6 (s, 2C, ArC-O), 137.8 (s, 2C, ArC), 135.5 (s, 2C, ArC), 126.7 (s, 2C, ArCH), 124.2 (s, 2C, ArCH), 119.86 (s, 1C, ArC), 54.5 (br s, 6C, Ar-CH_2_-N, N-CH), 35.5 (s, 2C, C(CH_3_)_3_), 34.2 (s, 2C, C(CH_3_)_3_), 32.4 (s, 6C, C(CH_3_)_3_), 30.2 (s, 6C, C(CH_3_)_3_), 29.7 (br s, 4C, CH_2_), 23.1 (br s, 2C, CH_2_), 17.1 (br s, 4C, CH_3_). Anal. Calcd (Found) for C_44_H_72_N_2_O_2_Zn: C, 72.75 (71.89); H, 9.99 (10.30); N, 3.86 (3.77)%.

#### Appendix A.1.3. (L^dmp^)_2_Mg

The synthesis of (L^dmp^)_2_Mg proceeds in the same manner as for (L^dmp^)_2_Zn using di-*n*-butyl-magnesium instead of diethylzinc. Yield 83% (0.57 g, 0.83 mmol). ^1^H NMR (500 MHz, C_6_D_6,_ RT) δ: Major form: 7.61 (d, *J*_HH_ = 2.6 Hz, 2H, ArCH), 7.11 (d, *J*_HH_ = 2.6 Hz, 2H, ArCH), 4.10 (br s, 2H, Ar-CH_2_-N), 3.88 (d, *J*_HH_ = 13.0 Hz, 2H, Ar-CH_2_-N), 3.65 (br s, 2H, N-CH), 3.21 (br s, 2H, N-CH), 1.71 (s, 18H, C(CH_3_)_3_), 1.50 (s, 18H, C(CH_3_)_3_), 1.99–1.76 (m, 4H, CH_2_), 1.43–1.38 (m, 2H, CH_2_), 1.30–1.20 (m, 4H, CH_2_), 1.18–1.08 (m, 2H, CH_2_), 0.98 (d, *J*_HH_ = 5.7 Hz, 6H, CH_3_), 0.88 (d, *J*_HH_ = 5.7 Hz, 6H, CH_3_); Minor form (selected chemical shifts): 7.62 (d, *J*_HH_ = 2.6 Hz, 1H, ArCH), 7.09 (d, *J*_HH_ = 2.6 Hz, 1H, ArCH), 3.98 (d, *J*_HH_ = 12.9 Hz, 2H, Ar-CH_2_-N), 3.92 (d, *J*_HH_ = 12.9 Hz, 2H, Ar-CH_2_-N), 2.98 (br s, 2H, N-CH), 2.92 (br s, 2H, N-CH), 1.72 (s, 18H, C(CH_3_)_3_), 1.51 (s, 18H, C(CH_3_)_3_), 1.04 (d, *J*_HH_ = 6.0 Hz, 6H, CH_3_), 0.84 (d, *J*_HH_ = 6.0 Hz, 6H, CH_3_). ^13^C NMR (126 MHz, C_6_D_6_, RT) δ: 163.0 (s, 2C, ArC-O), 137.1 (s, 2C, ArC), 135.3 (s, 2C, ArC), 126.5 (s, 2C, ArCH), 124.5 (s, 2C, ArC), 124.0 (s, 2C, ArCH), 59.2 (br s, 4C, N-CH), 52.4 (br s, 2C, Ar-CH_2_-N), 35.4 (s, 2C, C(CH_3_)_3_), 34.2 (s, 2C, C(CH_3_)_3_), 32.4 (s, 6C, C(CH_3_)_3_), 30.1 (s, 6C, C(CH_3_)_3_), 29.2 (br s, 4C, CH_2_), 23.8 (br s, 2C, CH_2_), 16.2 (br s, 4C, CH_3_). Anal. Calcd (Found) for C_44_H_72_N_2_O_2_Mg: C, 77.11 (76.79); H, 10.59 (10.83); N, 4.09 (3.89)%.

#### Appendix A.1.4. Representative Procedure for Solution Polymerization

The solution of (L^dmp^)_2_M in CH_2_Cl_2_ (20 mL) was placed in a Schlenk flask, and next, *L*-LA and an appropriate dye compound with hydroxyl group (ROH) were added simultaneously. The fixed molar ratio of a metal center [M] to *L*-LA and dye: [M]/*L*-LA/ROH = 1/n/1. The resulted solution was stirred at room temperature for a prescribed time monitored by ^1^H NMR spectroscopy.

Representative procedure for (L^dmp^)_2_Zn/Disperse Red 1: [Zn]/*L*-LA/DR1 = 1/100/1; (L^dmp^)_2_Zn (0.036 g, 0.05 mmol), *L*-LA (0.72 g, 5.00 mmol), DR1 (0.016 g, 0.05 mmol), time 5 min. The conversion was determined while observing ^1^H NMR resonances of the polymer and monomer by dissolving the precipitates in C_6_D_6_. After reaction was completed, an excess of hexanes was added to the reaction mixture. Filtration and vacuum drying yielded a red polymer. The resulting solid was dissolved in dichloromethane, and the polymer was precipitated with excess cold *n*-hexane. The polymer was collected by filtration, washed with methanol to remove unreacted monomer, and dried under reduced pressure. The reaction mixtures were prepared in a glovebox; then, subsequent operations were performed by means standard Schlenk techniques.

#### Appendix A.1.5. PLA-10-DR1

The synthesis was performed with the use of *L*-LA/(L^dmp^)_2_Zn/DR1 in the ratio 1/10/1. Yield 77% (0.68 g, 0.39 mmol). ^1^H NMR (500 MHz, C_6_D_6_) δ = 8.11–8.07 (m, 2H, ArCH), 7.96–7.92 (m, 2H, ArCH), 7.74–7.70 (m, 2H, ArCH), 6.46–6.42 (m, 2H, ArCH), 5.12–5.07 (m, 2H, CH), 5.04 (q, *J*_HH_ = 7.0 Hz, 16H, CH), 4.89 (q, *J*_HH_ = 7.1 Hz, 1H, CH), 4.12 (p, *J*_HH_ = 6.7 Hz, 1H, CH), 3.88–3.78 (m, 2H, NCH_2_), 3.05–2.87 (m, 2H, OCH_2_), 2.80 (q, *J*_HH_ = 7.0 Hz, 2H, NCH_2_), 2.49 (d, *J*_HH_ = 5.8 Hz, 1H, OH), 1.41 (d, *J*_HH_ = 7.1 Hz, 3H, CH_3_), 1.39 (d, *J*_HH_ = 7.1 Hz, 3H, CH_3_), 1.37 (d, *J*_HH_ = 7.1 Hz, 3H, CH_3_), 1.33 (d, *J*_HH_ = 7.1 Hz, 48H,, CH_3_), 1.06 (d, *J*_HH_ = 7.1 Hz, 3H, CH_3_), 0.70 (t, *J*_HH_ = 7.0 Hz, 3H, CH_3_). ^13^C NMR (126 MHz, C_6_D_6_) δ = 175.4 (s, 1C, CO), 170.1 (s, 1C, CO), 170.0 (s, 2C, CO), 169.9 (16C, CO), 156.7 (s, 1C, ArCNN), 151.2 (s, 1C, ArCN), 148.0 (s, 1C, ArNO_2_), 144.6 (s, 1C, ArCNN), 126.6 (s, 2C, ArCH), 124.8 (s, 2C, ArCH), 122.8 (s, 2C, ArCH), 111.8 (s, 2C, ArCH), 69.5 (s, 2C, CH), 69.4 (s, 16C, CH), 69.3 (s, 1C, CH), 66.9 (s, 1C, CH), 62.3 (s, NCH_2_), 48.3 (s, 1C, OCH_2_), 45.2 (s, 1C, NCH_2_), 20.8 (s, 1C, CH_3_), 16.7 (s, 1C, CH_3_), 16.6 (s, 1C, CH_3_), 16.5 (s, 16C, CH_3_), 16.4 (s, 2C, CH_3_), 12.0 (s, 1C, CH_3_).

#### Appendix A.1.6. PLA-10-DR13

The synthesis was performed with the use of *L*-LA/(L^dmp^)_2_Zn/DR13 in the ratio 1/10/1. Yield 84% (0.75 g, 0.42 mmol). ^1^H NMR (500 MHz, C_6_D_6_) δ = 8.13–8.08 (m, 2H, ArCH), 8.07 (d, *J*_HH_ = 2.4 Hz, 1H, ArCH), 7.65 (dd, *J*_HH_ = 8.9, 2.4 Hz, 1H, ArCH), 7.50 (d, *J*_HH_ = 8.9 Hz, 1H, ArCH), 6.40–6.36 (m, 2H, ArCH), 5.12–5.06 (m, 2H, CH), 5.04 (q, *J*_HH_ = 7.1 Hz, 16H, CH), 4.88 (q, *J*_HH_ = 7.1 Hz, 1H, CH), 4.12 (p, *J*_HH_ = 6.7 Hz, 1H, CH), 3.88–3.77 (m, 2H, NCH_2_), 3.02–2.86 (m, 2H, OCH_2_), 2.80 (q, *J*_HH_ = 7.0 Hz, 2H, NCH_2_), 2.51 (d, *J*_HH_ = 5.8 Hz, 1H, OH), 1.41 (d, *J*_HH_ = 7.1 Hz, 3H, CH_3_), 1.40–1.36 (m, 6H, CH_3_), 1.33 (d, *J*_HH_ = 7.1 Hz, 48H,, CH_3_), 1.06 (d, *J*_HH_ = 7.1 Hz, 3H, CH_3_), 0.69 (t, *J*_HH_ = 7.0 Hz, 3H, CH_3_). ^13^C NMR (126 MHz, C_6_D_6_) δ = 175.4 (s, 1C, CO), 170.1 (s, 1C, CO), 170.0 (s, 2C, CO), 169.9 (16C, CO), 152.8 (s, 1C, ArCNN), 151.5 (s, 1C, ArCN), 148.0 (s, 1C, ArNO_2_), 145.2 (s, 1C, ArCNN), 134.3 (s, 1C, ArCCl), 127.2 (s, 2C, ArCH), 126.3 (s, 1C, ArCH), 122.6 (s, 1C, ArCH) 117.9 (s, 1C, ArCH), 111.9 (s, 2C, ArCH), 69.4 (s, 18C, CH), 69.3 (s, 1C, CH), 66.9 (s, 1C, CH), 62.2 (s, NCH_2_), 48.3 (s, 1C, OCH_2_), 45.3 (s, 1C, NCH_2_), 20.8 (s, 1C, CH_3_), 16.7 (s, 1C, CH_3_), 16.6 (s, 1C, CH_3_), 16.5 (s, 16C, CH_3_), 16.4 (s, 2C, CH_3_), 12.0 (s, 1C, CH_3_).
