# Supplementary material for: Lastingly Colored Polylactide Synthesized by Dye-Initiated Polymerization
Source: Polymers (Basel). 2020 Aug 31;12(9):1980. doi: 10.3390/polym12091980 (PMC7563163; doi:10.3390/polym12091980)
Supplement: Supplementary file 1 [file polymers-12-01980-s001.pdf]

Supporting Information

Lastingly coloured PLA synthesized by dye-initiated polymerization

Dawid Jędrzkiewicz [1], Sebastian Kowalczyk [2], Andrzej Plichta [2], Jolanta Ejfler,[1]*

[1] Faculty of Chemistry, University of Wroclaw, 14 Joliot-Curie Str., 50-383 Wrocław, Poland

[2] Faculty of Chemistry, Warsaw University of Technology, 3 Noakowskiego Str., 00-664 Warsaw, Poland

* jolanta.ejfler@chem.uni.wroc.pl

Table of Contents	Page
**Figure S1.** ^{1}H NMR of L^{dmp}-H in C_6D_6.	2
**Figure S2.** ^{13}C NMR of L^{dmp}-H in C_6D_6.	2
**Figure S3**. ^{1}H NMR of $(L^{dmp})_2Zn$ in C_6D_6.	3
**Figure S4.** ^{13}C NMR of $(L^{dmp})_2Zn$ in C_6D_6.	3
**Figure S5.** ^{1}H COSY of $(L^{dmp})_2Zn$ in C_6D_6.	4
**Figure S6.** ^{1}H NOESY of $(L^{dmp})_2Zn$ in C_6D_6.	4
**Figure S7**. ^{1}H NMR of $(L^{dmp})_2Mg$ in C_6D_6.	5
**Figure S8.** ^{13}C NMR of $(L^{dmp})_2Mg$ in C_6D_6.	5
**Figure S9.** ^{1}H COSY of $(L^{dmp})_2Mg$ in C_6D_6.	6
**Figure S10.** ^{1}H NOESY of $(L^{dmp})_2Mg$ in C_6D_6.	6
**Figure S11.** ^{1}H NMR of PLA-10-DR1 in C_6D_6.	7
**Figure S12.** ^{13}C NMR of PLA-10-DR1 in C_6D_6.	7
**Figure S13.** ^{1}H COSY of PLA-10-DR1 in C_6D_6.	8
**Figure S14.** ^{1}H NMR of PLA-10-DR13 in C_6D_6.	8
**Figure S15.** ^{13}C NMR of PLA-10-DR13 in C_6D_6.	9
**Figure S16.** ^{1}H COSY of PLA-10-DR13 in C_6D_6.	9
**Table S1.** Results of MALDI ToF on ROP of L-LA initiated by zinc and magnesium complexes with Disperse Red 1 (DR1) and Disperse Red 13 (DR13) as co-initiators.	10
**Figure S17.** MALDI ToF mass spectra of products no. 2.	11
**Figure S18.** MALDI ToF mass spectra of products no. 3.	11
**Figure S19.** MALDI ToF mass spectra of products no. 4.	12
**Figure S20.** MALDI ToF mass spectra of products no. 7.	12
**Figure S21.** MALDI ToF mass spectra of products no. 8.	13
**Figure S22.** MALDI ToF mass spectra of products no. 9.	13
**Figure S23.** MALDI ToF mass spectra of products no. 10.	14
**Figure S24.** MALDI ToF mass spectra of products no. 11.	14
**Table S2.** X-ray experimental data and refinement for $(L^{dmp})_2Zn$ and $(L^{dmp})_2Mg$.	15
**Table S3.** Selected bond distances and angles for $(L^{dmp})_2Zn$ and $(L^{dmp})_2Mg$.	16
**Supplementary literature**	16

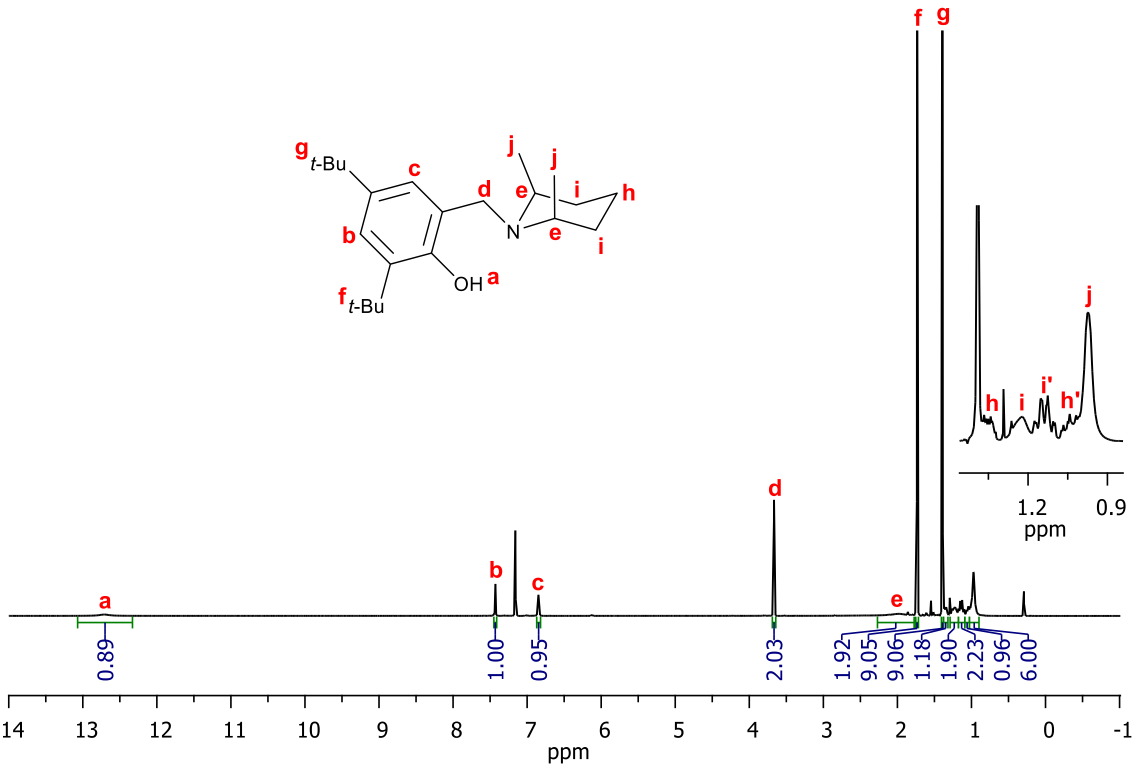

Figure S1. ^{1}H NMR of L^{dmp}-H in C_6D_6.

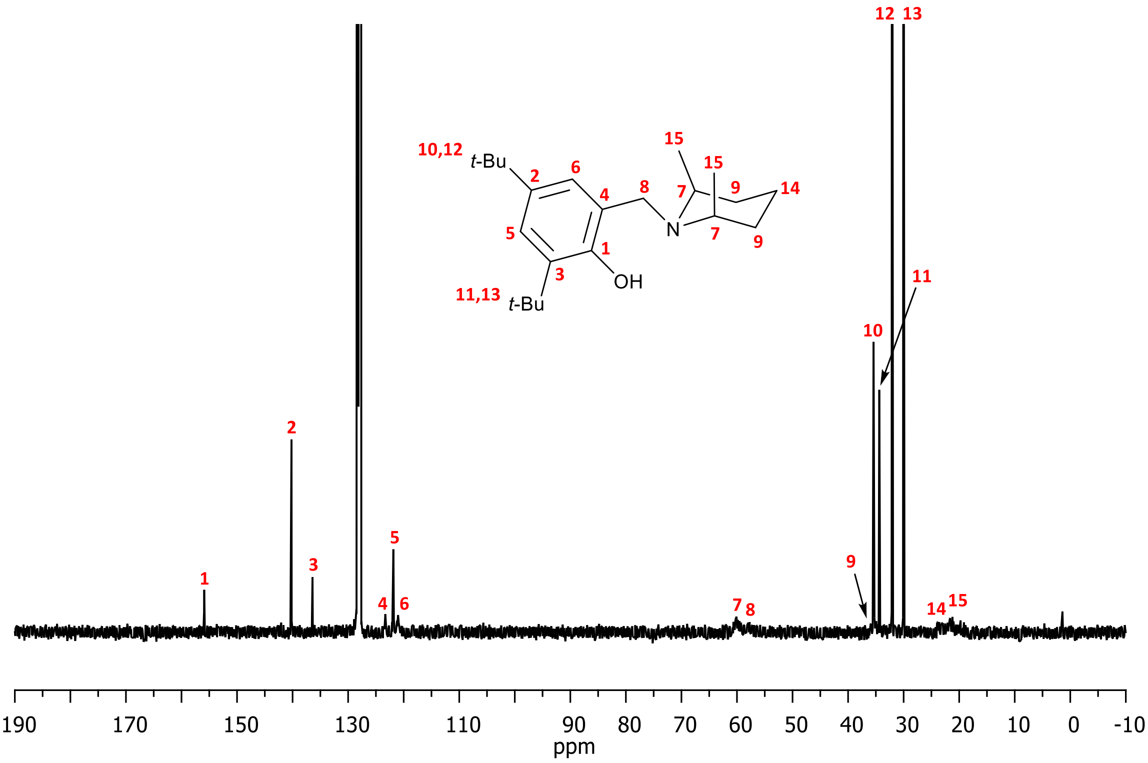

Figure S2. ^{13}C NMR of L^{dmp}-H in in C_6D_6.

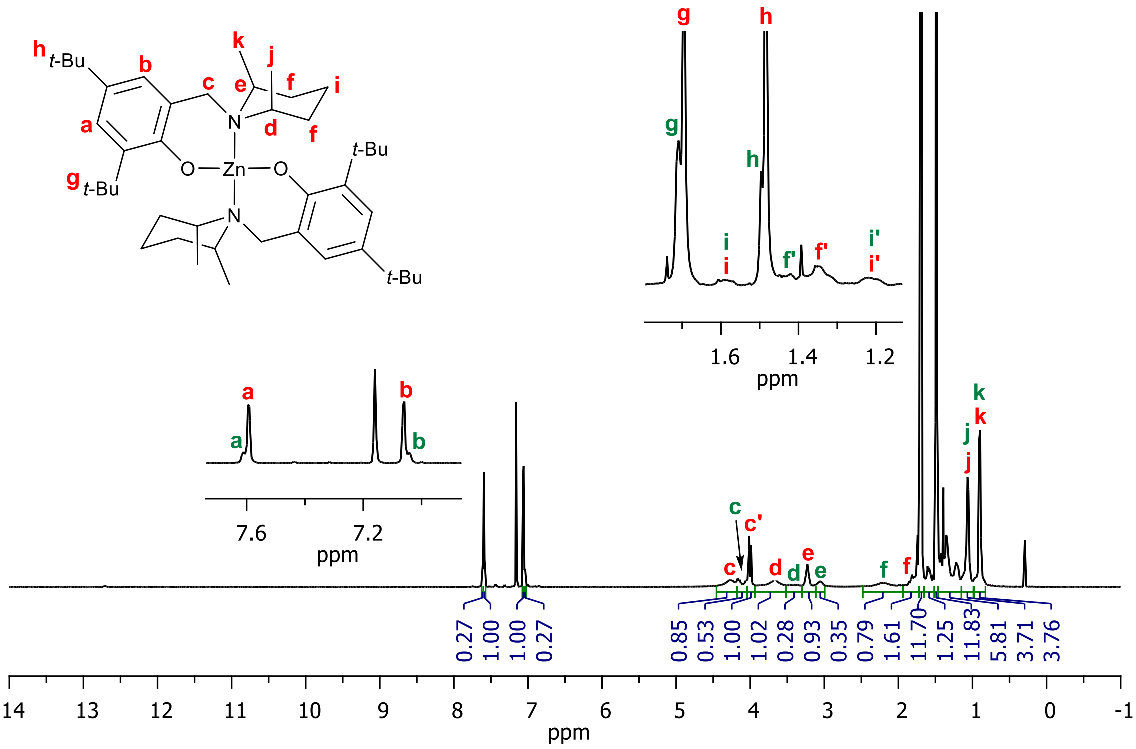

Figure S3. ^{1}H NMR of $(L^{dmp})_2Zn$ in C_6D_6.

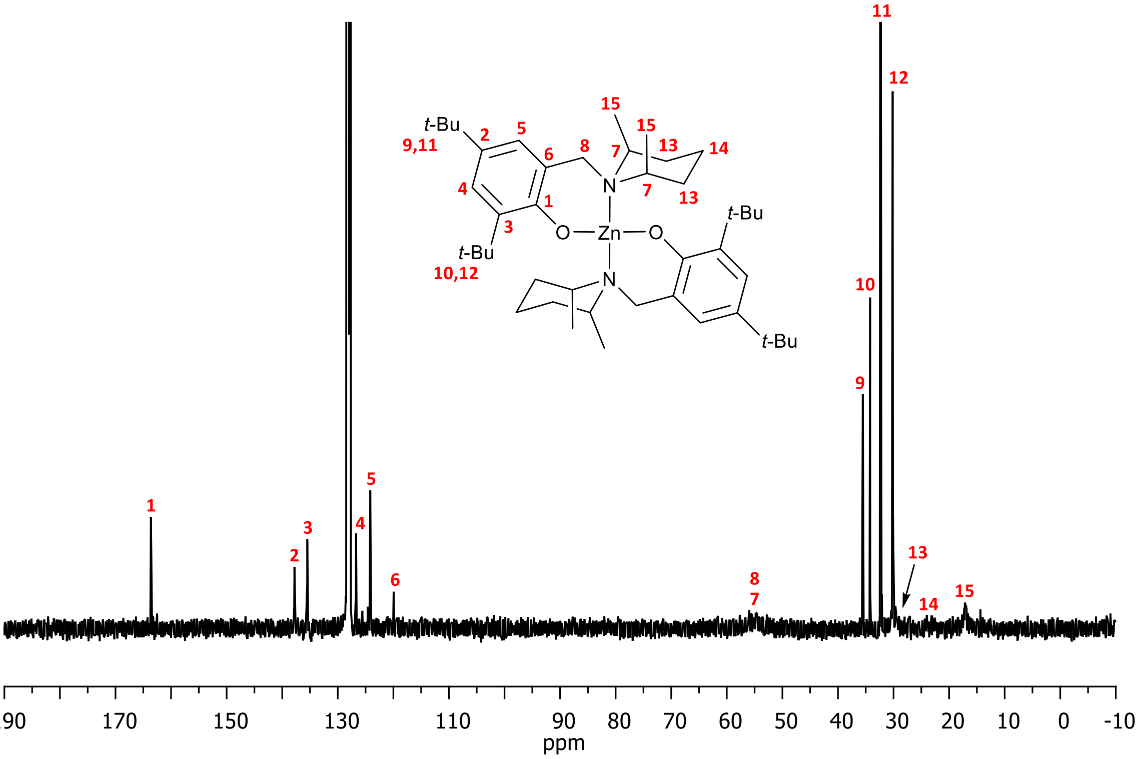

Figure S4. ^{13}C NMR of $(L^{dmp})_2Zn$ in C_6D_6.

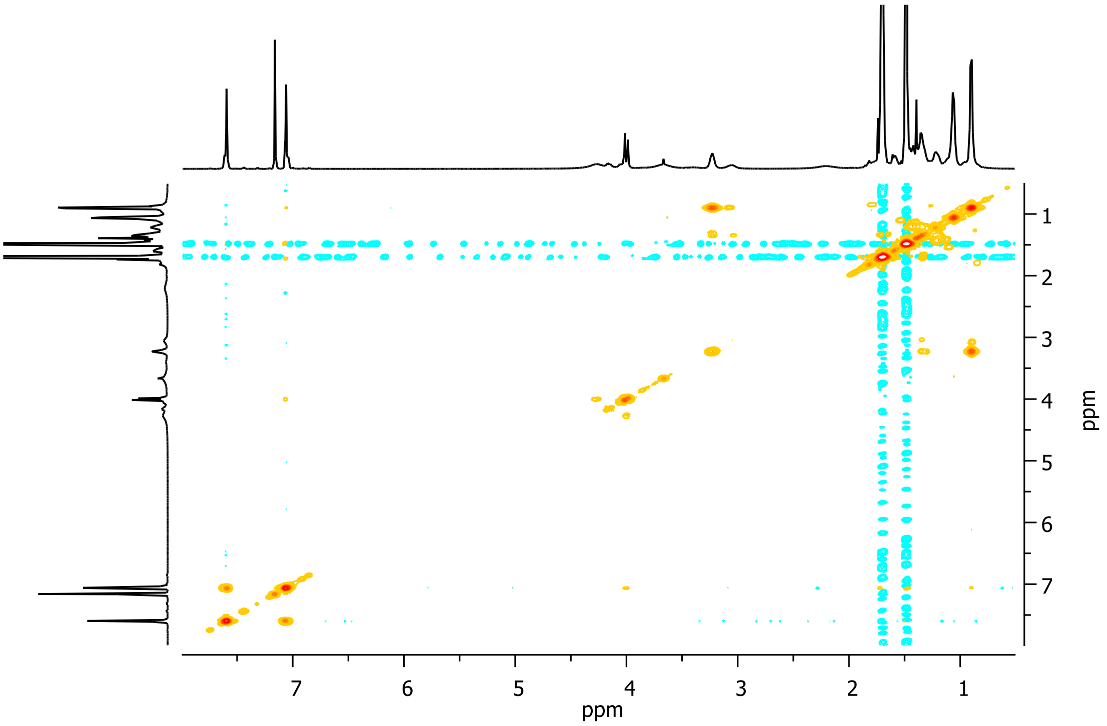

Figure S5. ^{1}H COSY of $(L^{dmp})_2Zn$ in C_6D_6.

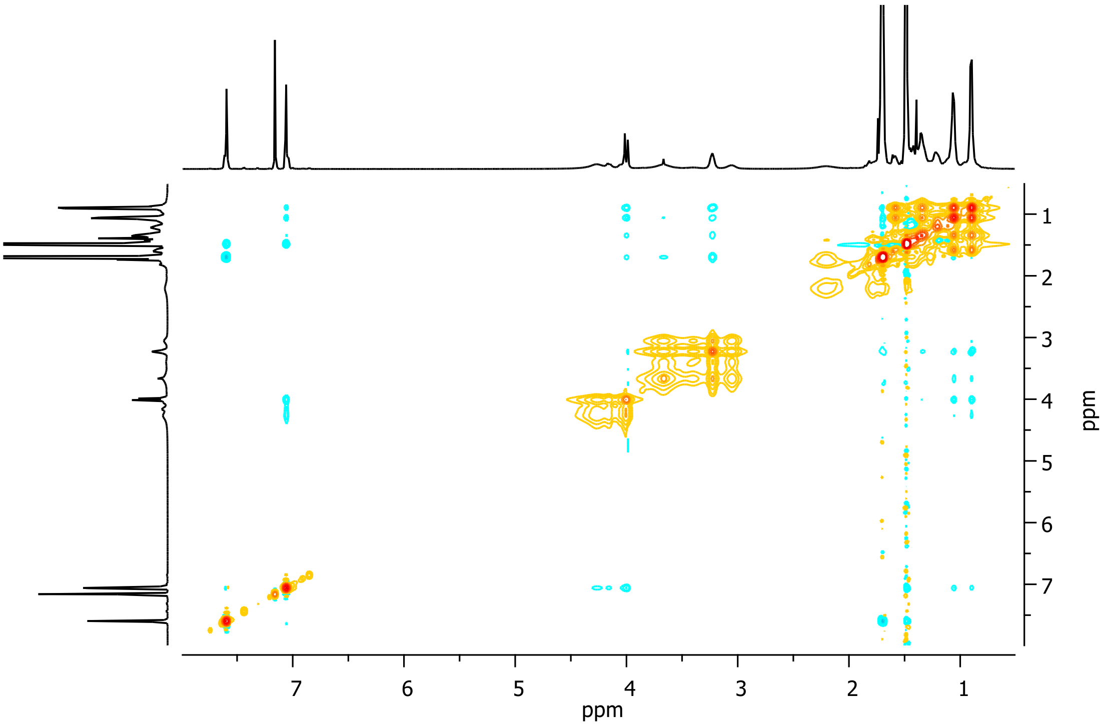

Figure S6. ^{1}H NOESY of $(L^{dmp})_2Zn$ in C_6D_6.

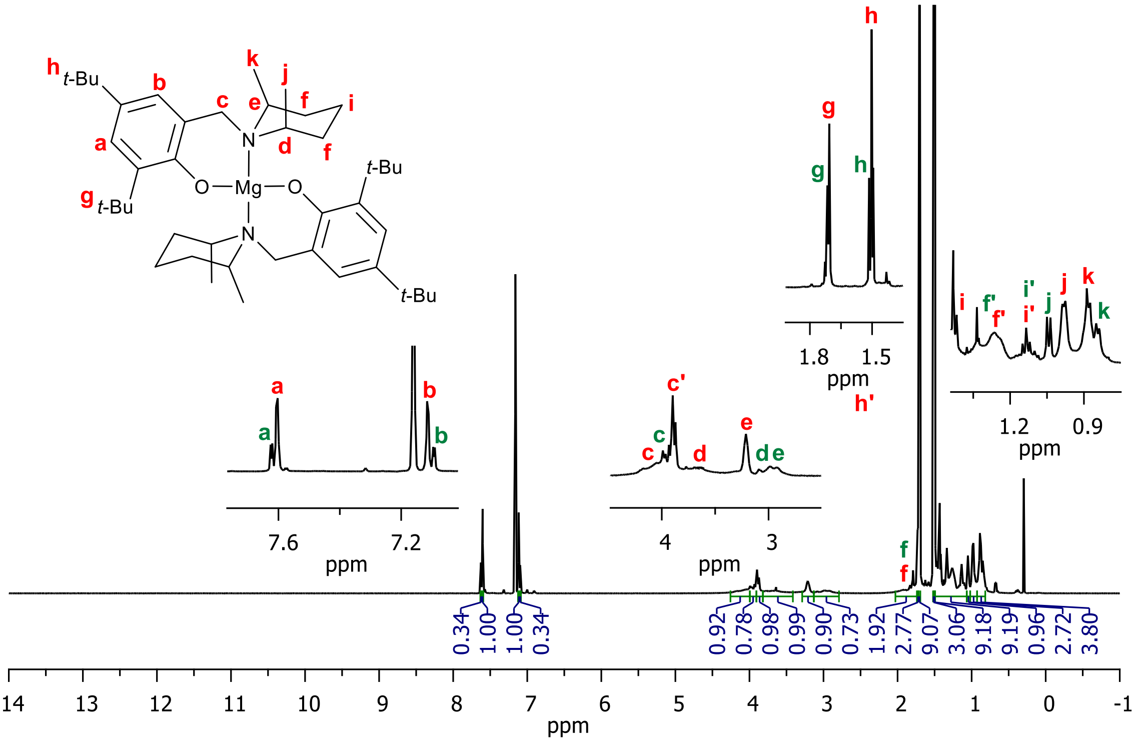

Figure S7. ^{1}H NMR of $(L^{dmp})_2Mg$ in C_6D_6.

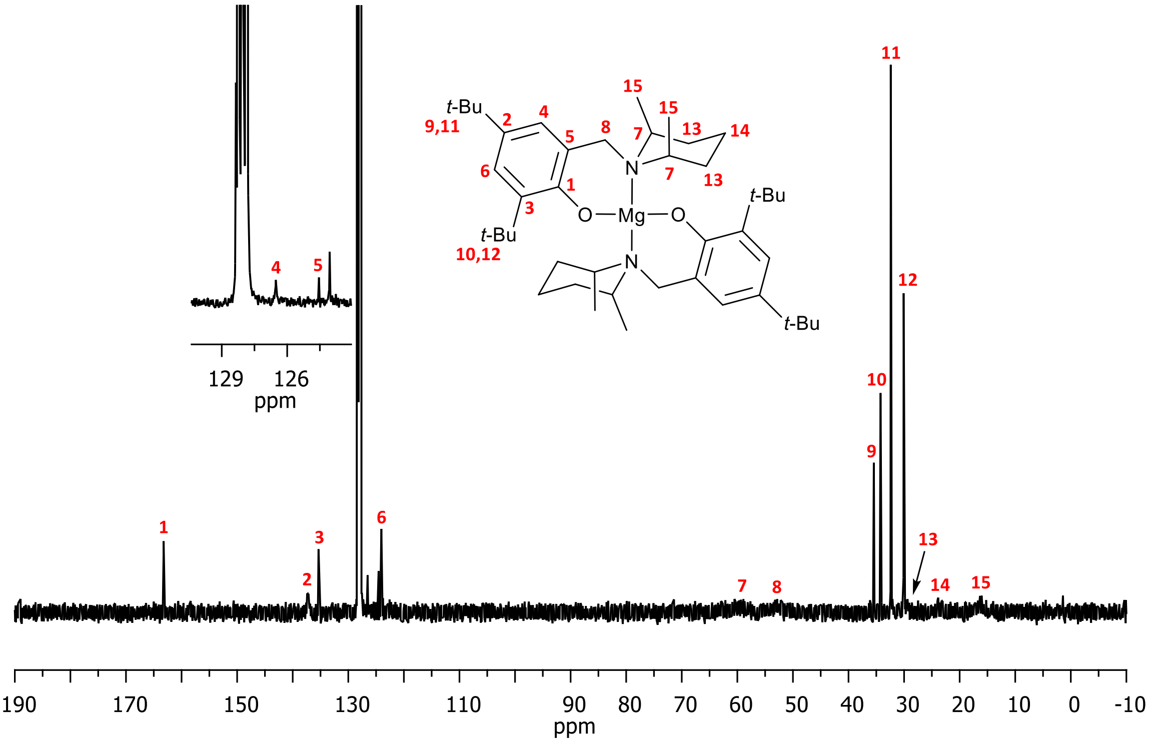

Figure S8. ^{13}C NMR of $(L^{dmp})_2Mg$ in C_6D_6.

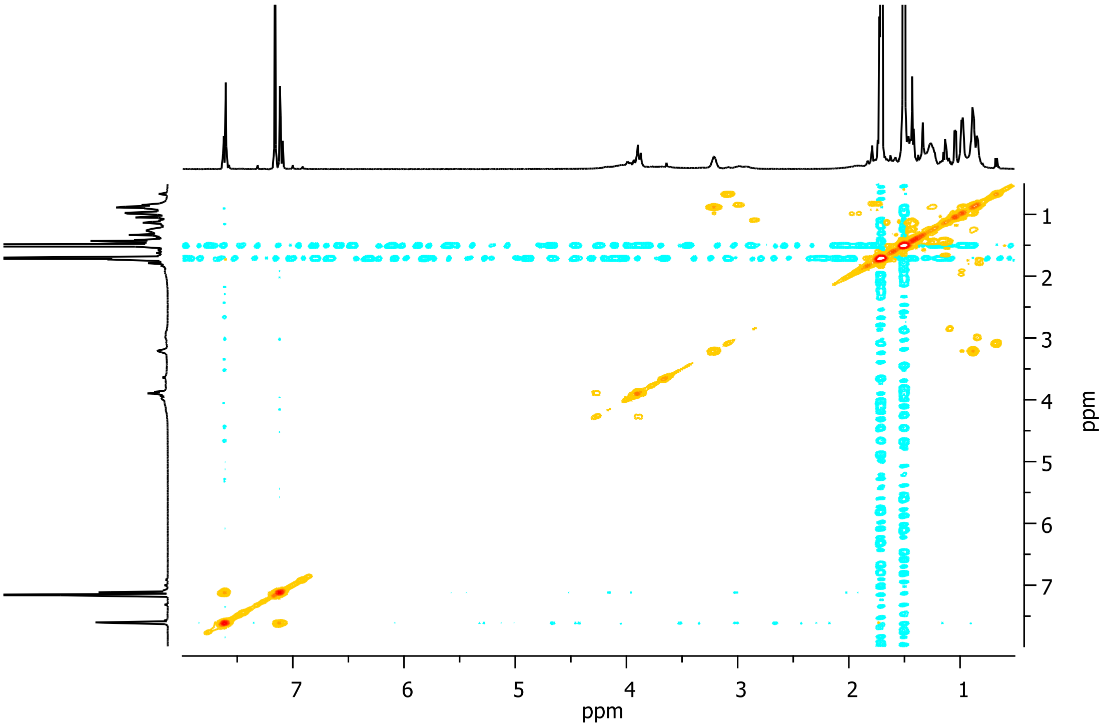

Figure S9. ^{1}H COSY of $(L^{dmp})_2Mg$ in C_6D_6.

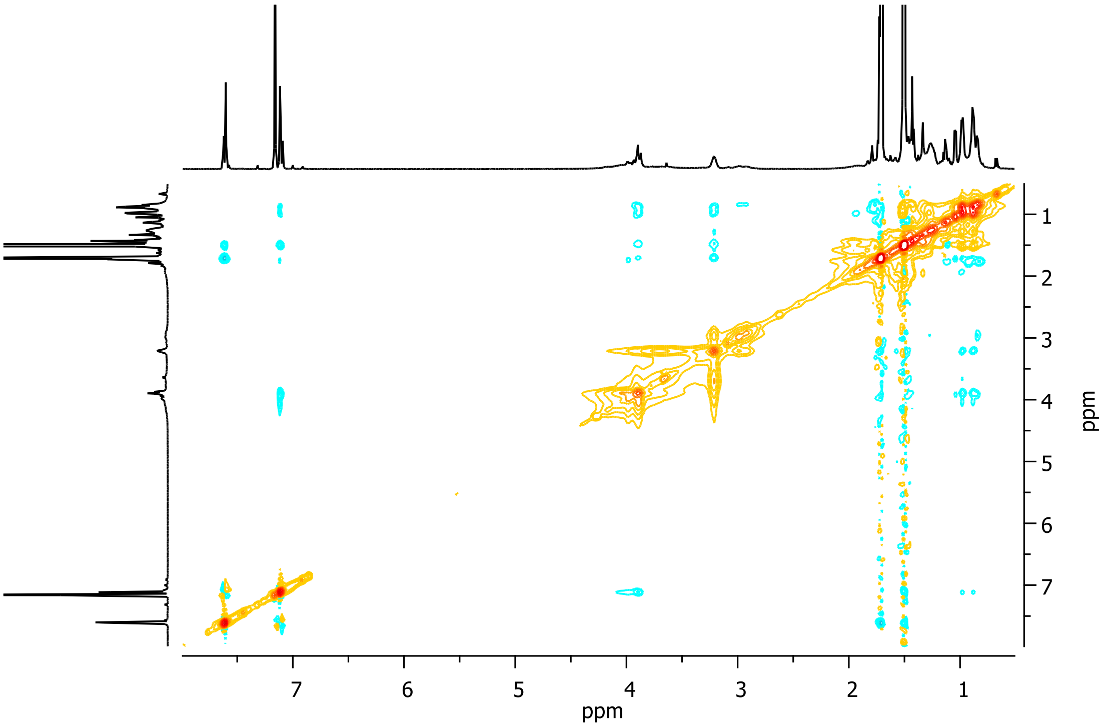

Figure S10. ^{1}H NOESY of $(L^{dmp})_2Mg$ in C_6D_6.

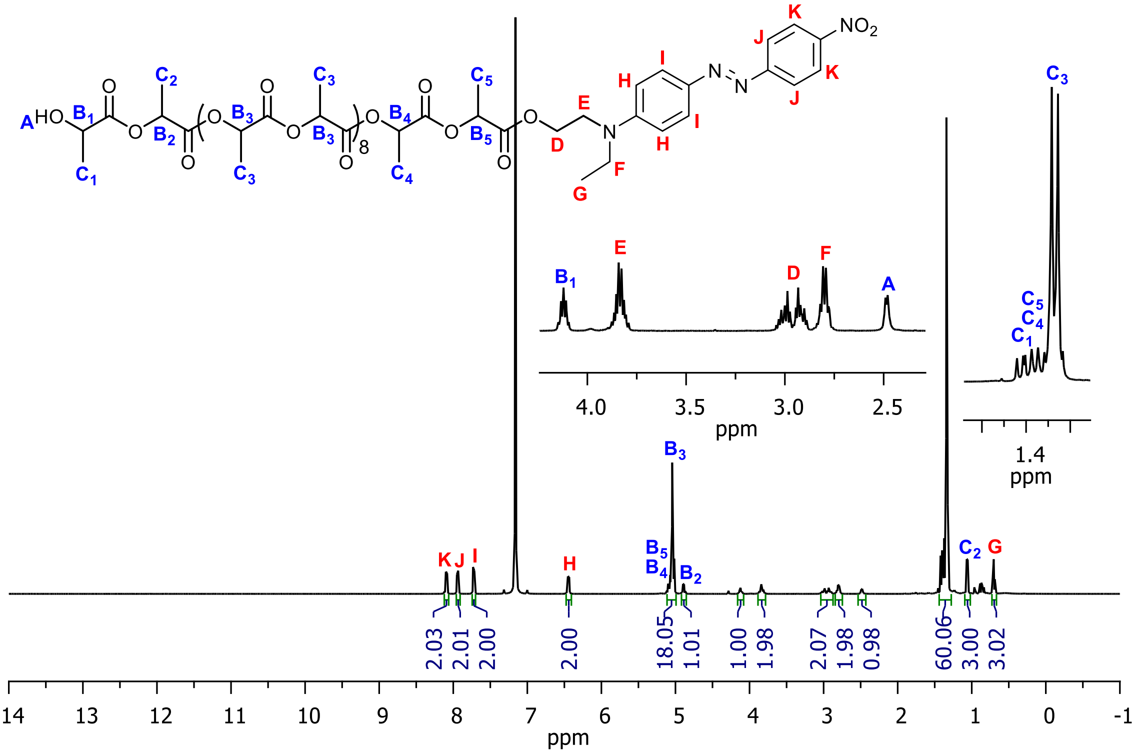

Figure S11. ^{1}H NMR of PLA-10-DR1 in C_6D_6.

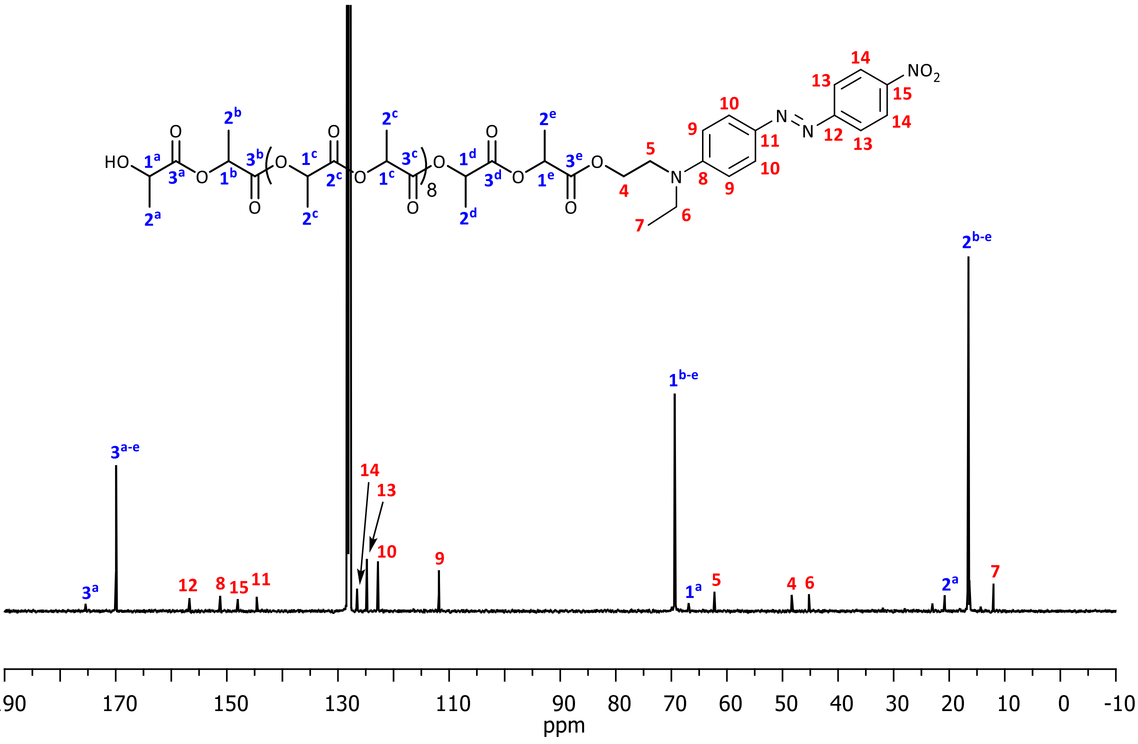

Figure S12. ^{13}C NMR of PLA-10-DR1 in C_6D_6.

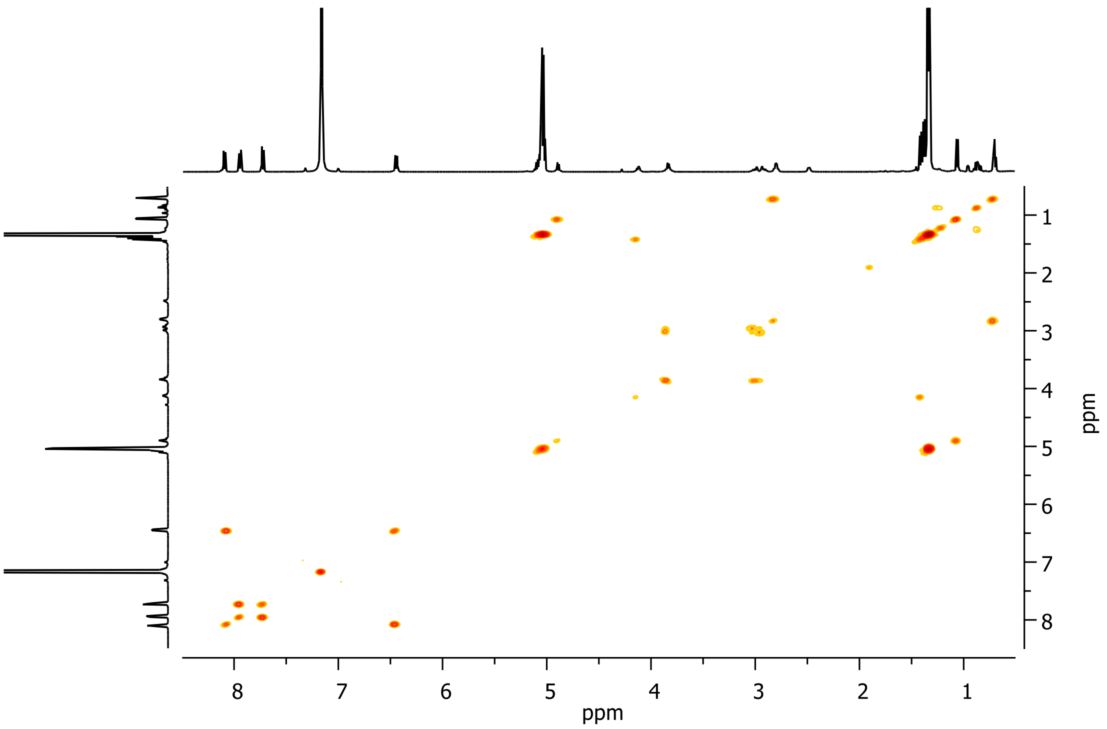

Figure S13. ^{1}H NOESY of PLA-10-DR1 in C_6D_6.

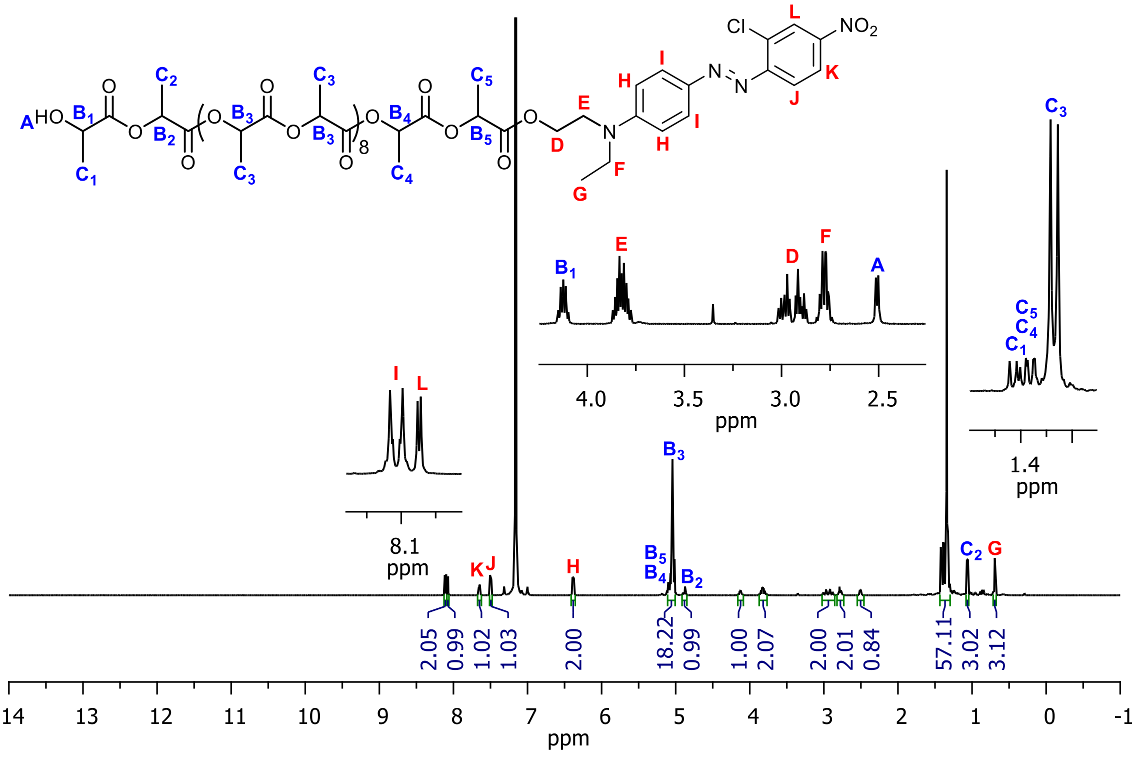

Figure S14. ^{1}H NMR of PLA-10-DR13 in C_6D_6.

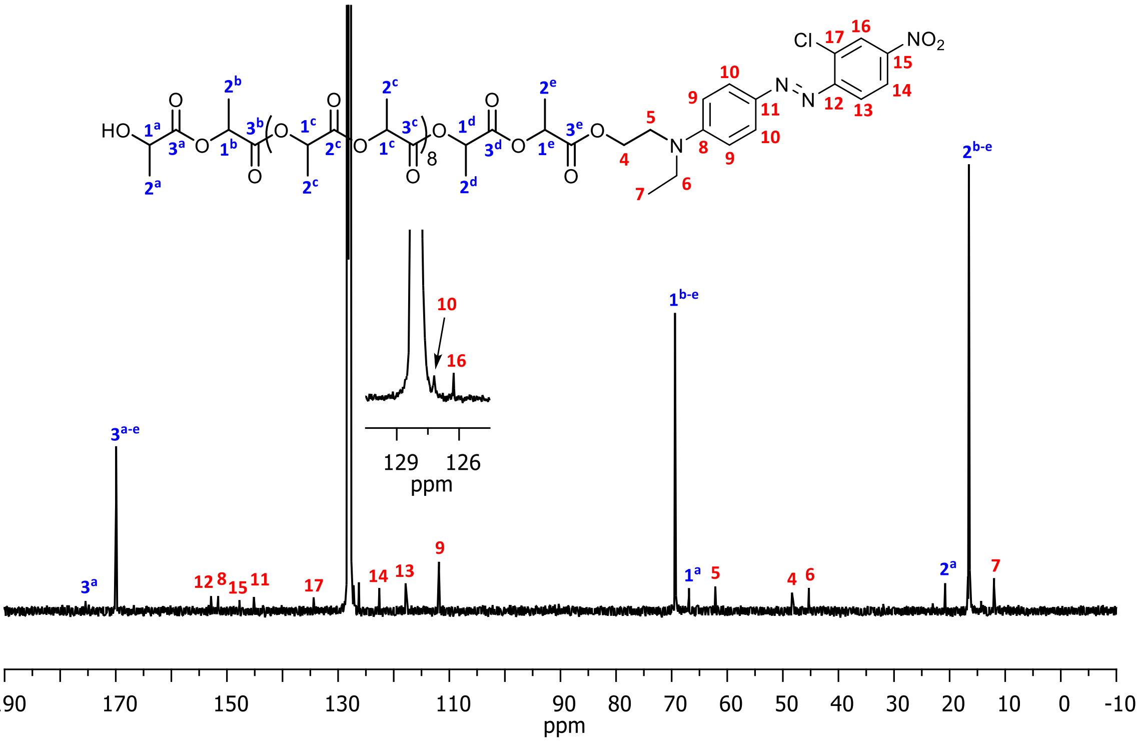

Figure S15. ^{13}C NMR of PLA-10-DR13 in C_6D_6.

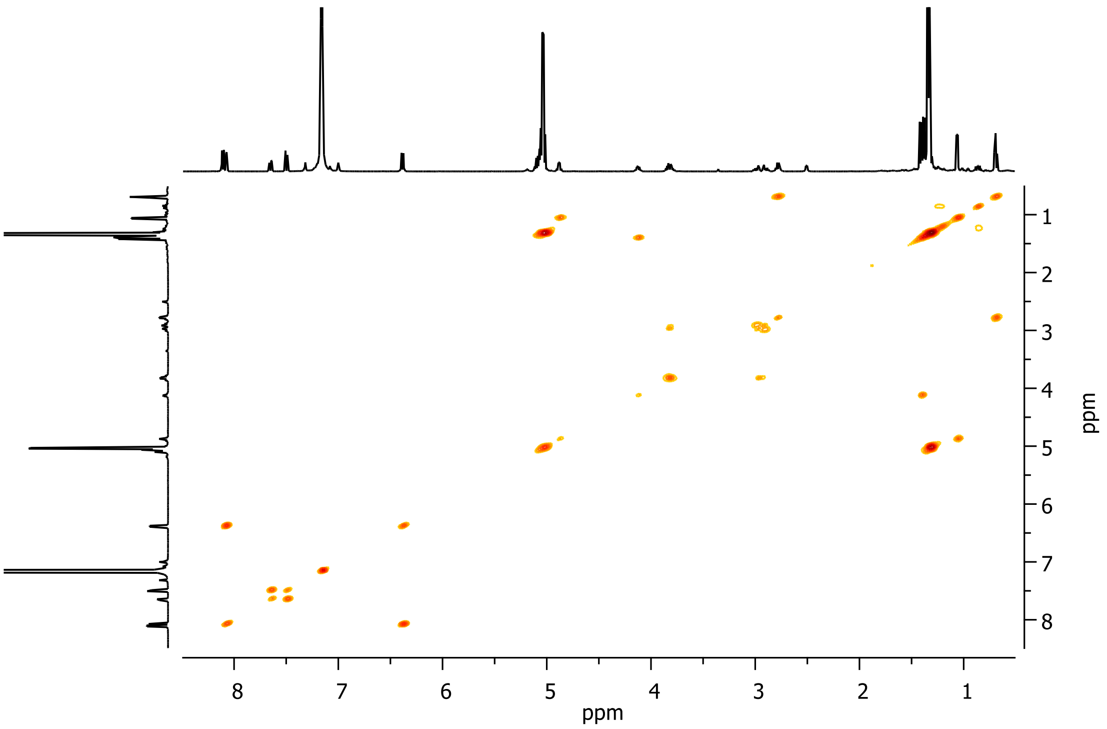

Figure S16. ^{1}H NOESY of PLA-10-DR1 in C_6D_6.

No.	Initiator [I]	Molar ratios[a]	ROH	End-group in the population	$M_{n,MALDI}$[b]	$Đ_{M,MALDI}$[b]	Fraction of number of molecules in population (%)[b]	even/odd numbers of lactic acid m.u.[b,c]
1	$(L_{dmp})_2Zn$	1/10/1	DR13	DR13	2420	1.12	62.1	e
					2770	1.13	36.6	o
				H-OH	1528	1.19	1.3	e
2	$(L_{dmp})_2Zn$	1/40/1	DR13	DR13	3563	1.14	52.3	e
					3449	1.15	41.7	o
				H-OH	2917	1.05	1.1	e
					2936	1.04	0.9	o
				-[d]	2530	1.07	1.9	e
					2533	1.07	2.1	o
3	$(L_{dmp})_2Zn$	1/100/1	DR13	DR13	3104	1.16	39.1	e
					3139	1.17	37.8	o
				-[d]	2517	1.10	11.4	e
					2508	1.10	11.7	o
4	$(L_{dmp})_2Zn$	1/200/1	DR13	DR13	2814	1.13	38.0	e
					2907	1.11	35.7	o
				-[d]	1866	1.05	12.8	e
					1837	1.05	13.5	o
6	$(L_{dmp})_2Zn$	1/10/1	DR1	DR1	2463	1.10	60.4	e
					2539	1.11	39.6	o
7	$(L_{dmp})_2Zn$	1/30/1	DR1	DR1	2825	1.19	48.7	e
					2803	1.20	48.9	o
				-[d]	2559	1.04	1.2	e
					2524	1.05	1.2	o
8	$(L_{dmp})_2Zn$	1/100/1	DR1	DR1	2742	1.16	44.4	e
					2733	1.16	44.7	o
				-[d]	2223	1.13	5.6	e
					2322	1.11	5.3	o
9	$(L_{dmp})_2Mg$	1/100/1	DR1	DR1	2852	1.18	41.9	e
					2870	1.18	39.9	o
				-[d]	2747	1.15	9.1	e
					2790	1.15	9.1	o
10	$(L_{dmp})_2Mg$	1/200/1	DR1	DR1	2826	1.10	43.0	e
					2829	1.10	43.0	o
				-[d]	1901	1.01	6.9	e
					1850	1.02	7.1	o
11	$(L_{dmp})_2Mg$	1/300/1	DR1	DR1	3539	1.10	29.6	e
					3545	1.09	29.0	o
				-[d]	3222	1.06	20.2	e
					3180	1.06	21.2	o

General remarks: $M_{n,MALDI}$ expressed in g/mol; remarks: [a] initial molar ratio of $[I]_0/[L\text{-LA}]_0/[ROH]_0$; [b] determined by MALDI ToF measurement; [c] 'e' stands for even and 'o' for odd numbers of lactic acid monomeric units in populations; [d] no end group – population of macrocyclic products.

Table S1. Results of MALDI ToF on ROP of *L*-LA initiated by zinc and magnesium complexes with Disperse Red 1 (DR1) and Disperse Red 13 (DR13) as co-initiators.

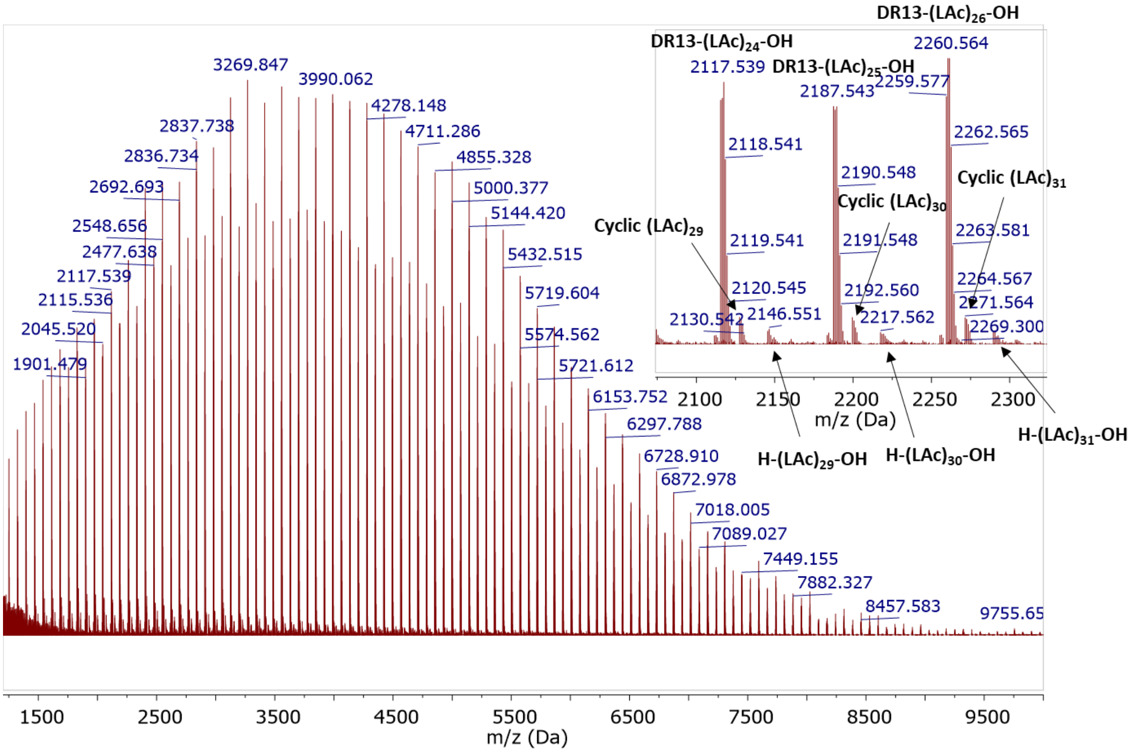

Figure S17. MALDI ToF mass spectra of products no. 2.

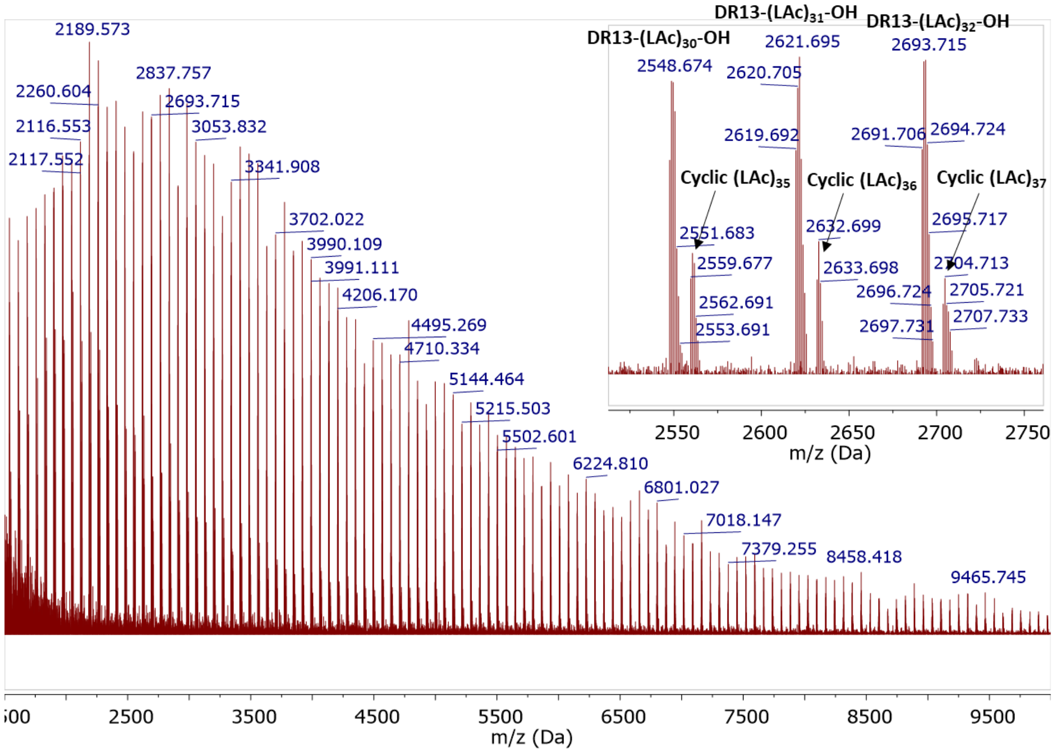

Figure S18. MALDI ToF mass spectra of products no. 3.

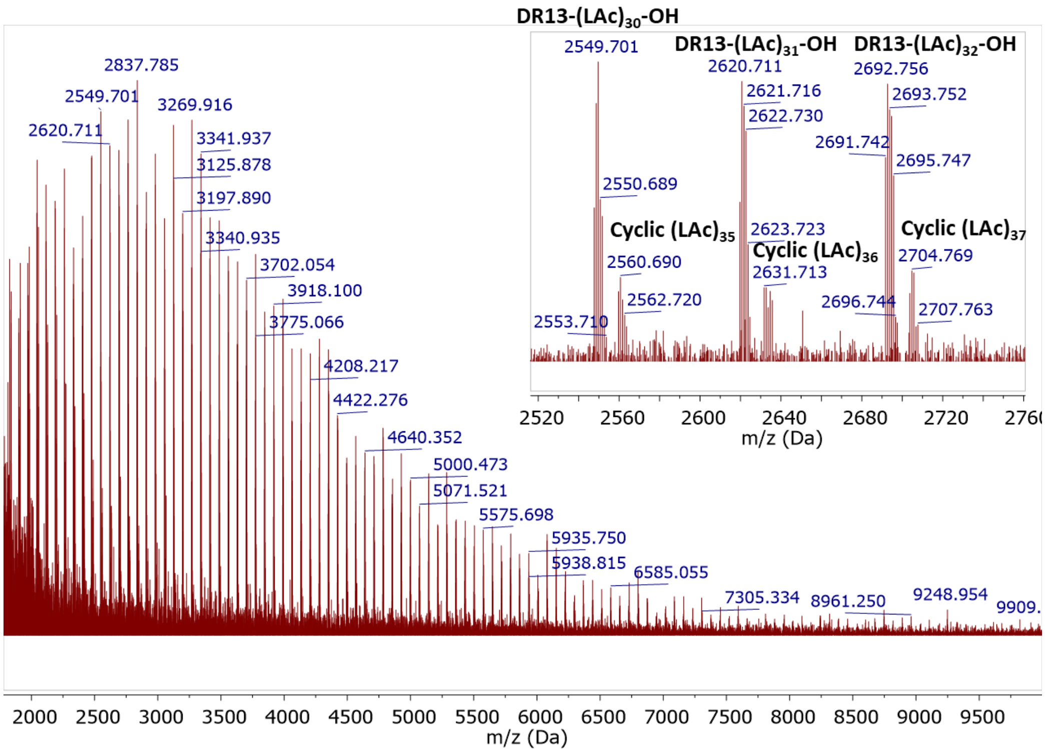

Figure S19. MALDI ToF mass spectra of products no. 4.

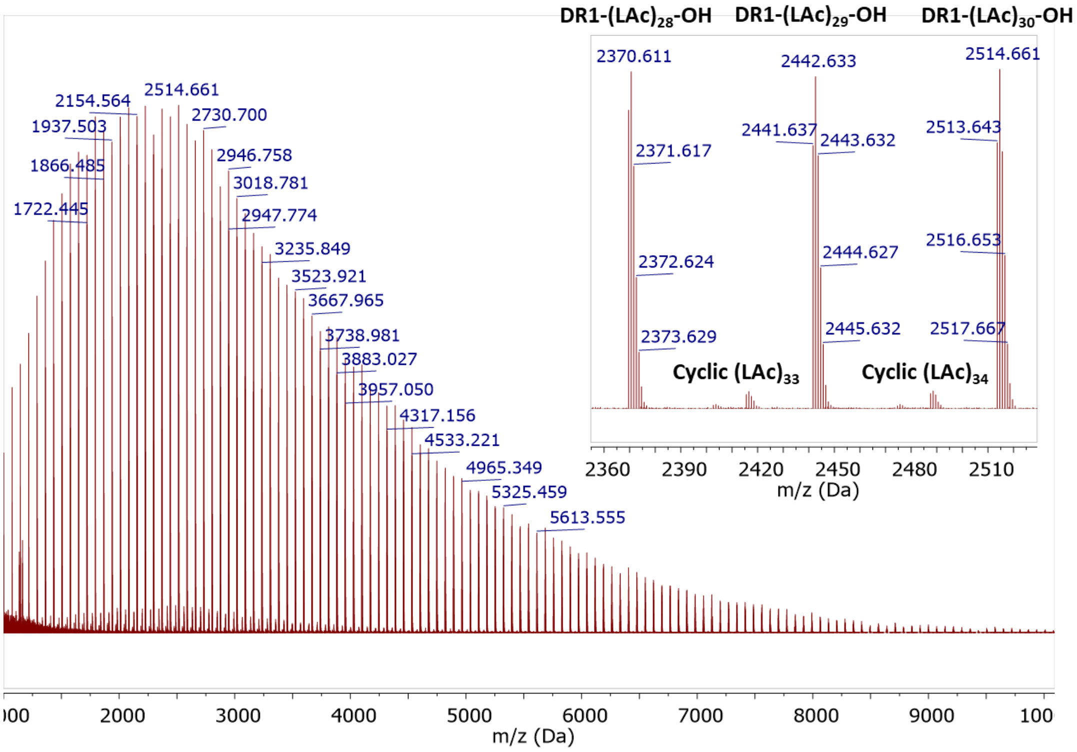

Figure S20. MALDI ToF mass spectra of products no. 7.

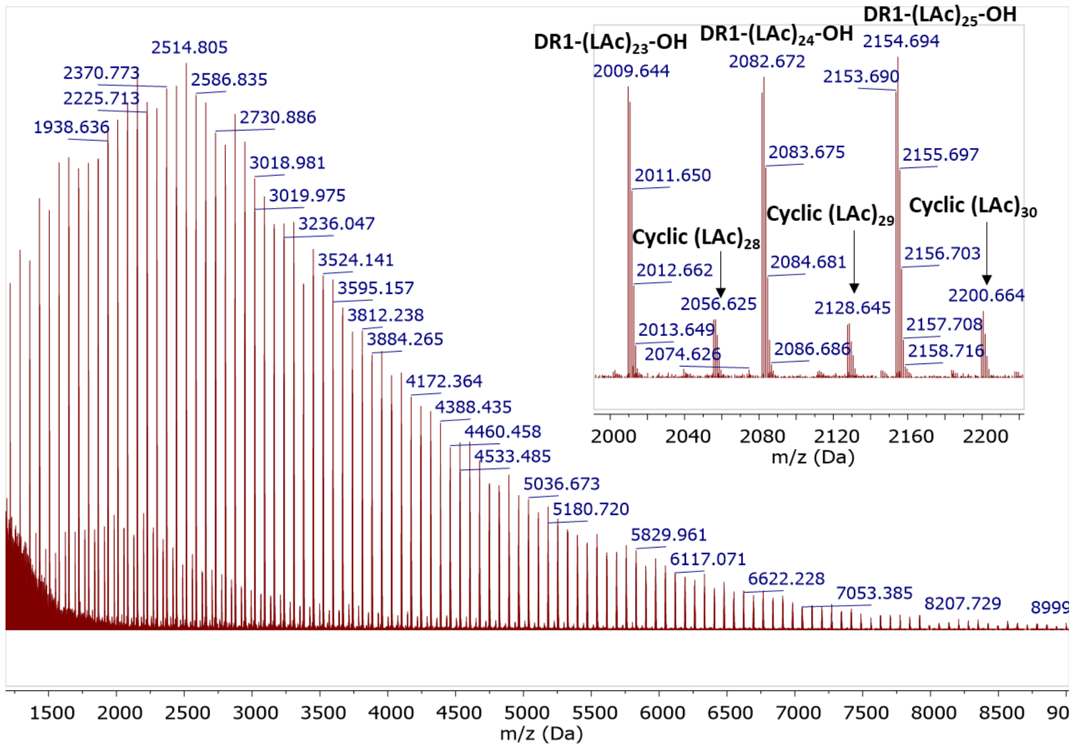

Figure S21. MALDI ToF mass spectra of products no. 8.

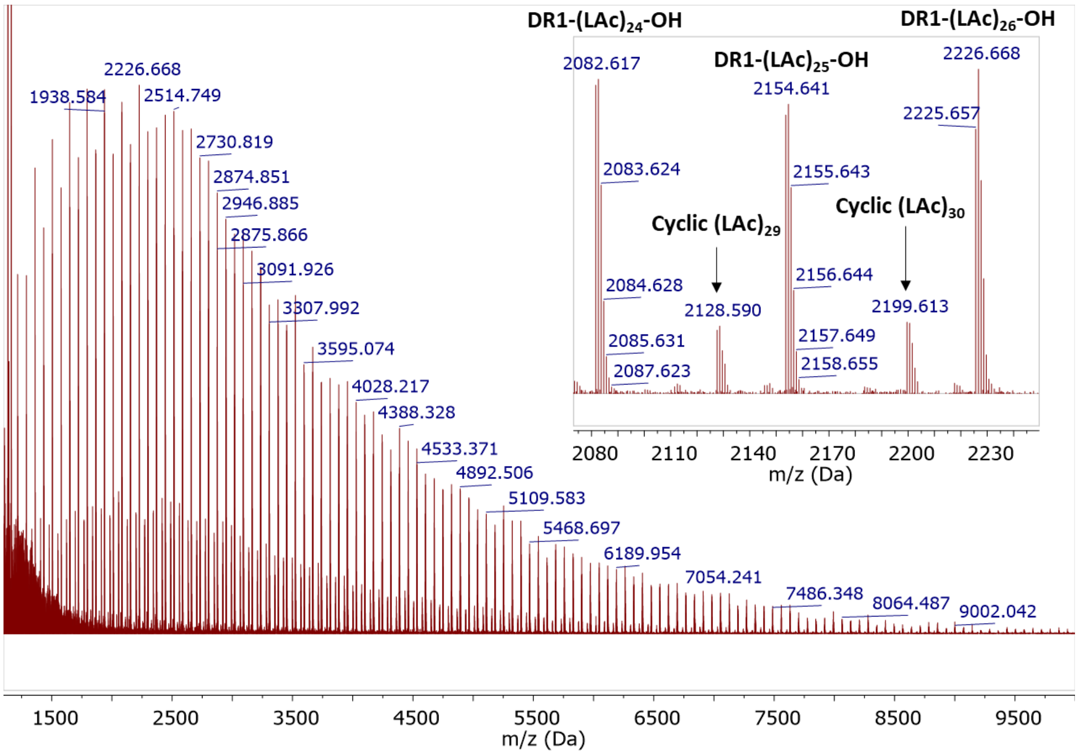

Figure S22. MALDI ToF mass spectra of products no. 9.

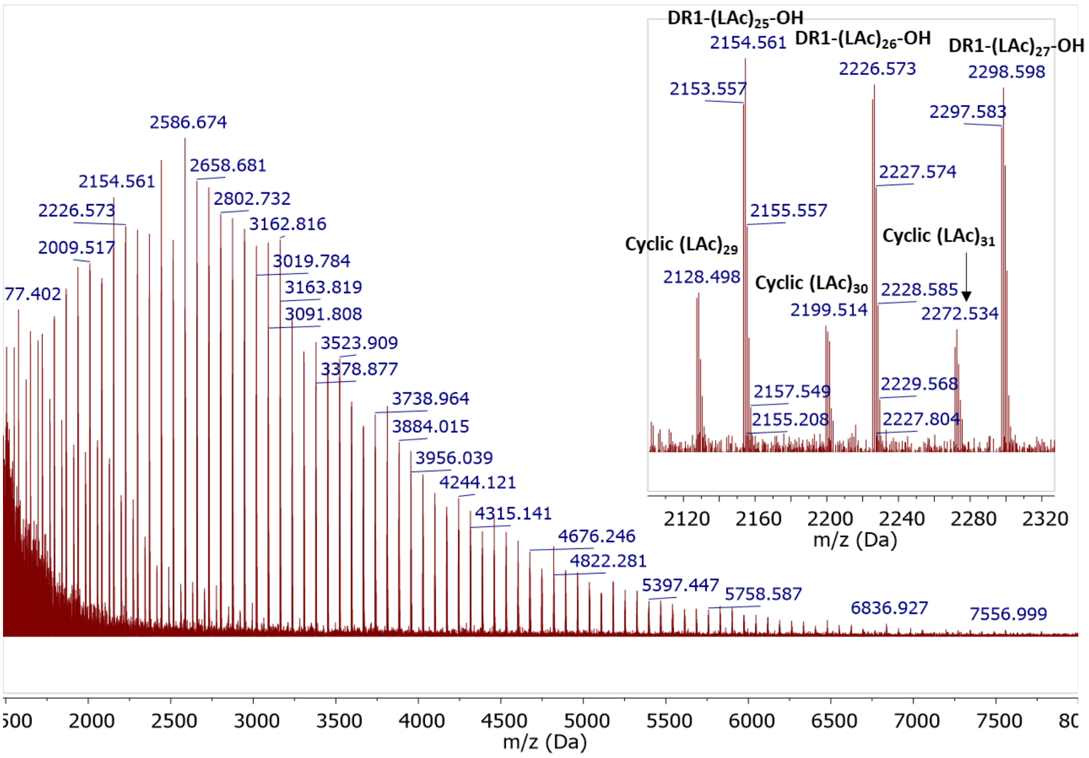

Figure S23. MALDI ToF mass spectra of products no. 10.

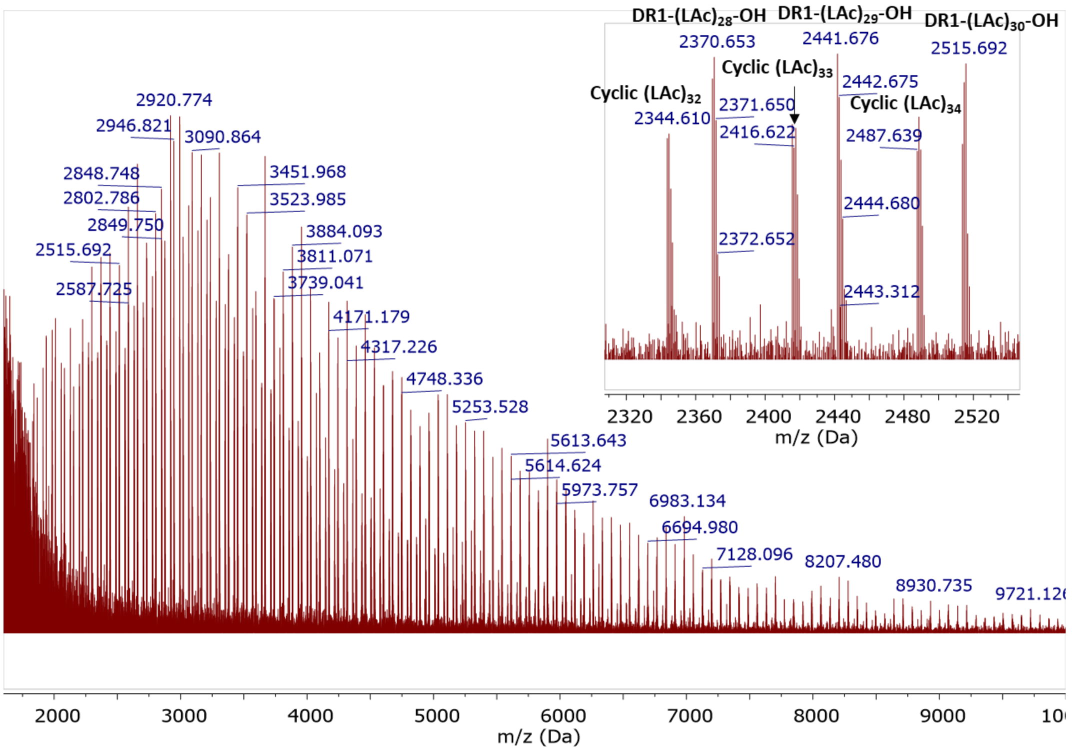

Figure S24. MALDI ToF mass spectra of products no. 11.

	$(L^{dmp})_2Zn$	$(L^{dmp})_2Mg$
Empirical formula	$C_{44}H_{72}N_2O_2Zn$	$C_{44}H_{72}N_2O_2Mg$
Formula weight	726.40	685.34
Crystal system	Monoclinic	Monoclinic
Space group	*I2/a*	*I2/a*
a (Å)	18.683(7)	18.927(6)
b (Å)	8.782(3)	8.782(3)
c (Å)	26.058(9)	26.190(8)
α (°)	90	90
β (°)	110.34(4)	110.64(4)
γ (°)	90	90
V (Å^3)	4009(3)	4074(2)
Z	4	4
Crystal description	Block, colourless	Needle, colourless
Crystal size (mm)	0.29 × 0.18 × 0.11	0.36 × 0.10 × 0.07
d_{calc} (g/cm^3)	1.204	1.117
μ (mm^{-1})	0.65	0.08
F(000)	1584	1512
Diffractometer	Xcalibur, CCD Ruby	Xcalibur, CCD Ruby
λ (Å)	0.71073 (Mo)	0.71073 (Mo)
T (K)	100	110
Θ min/max (°)	1.7/28.7	1.7/25.5
h, k, l min/max	-25/20, -8/11, -33/35	-22/17, -10/6, -31/23
Reflections collected	8940	6805
Independent reflections	4513	3712
Reflections [I>2σ(I)]	3430	1953
R (int.)	0.035	0.066
data/restraints/params	4513/264/327	3712/230/327
$R[F^2 > 2\sigma(F^2)]$	0.042	0.065
$wR(F^2)$	0.083	0.146
GooF	1.04	0.993
$\Delta\rho_{max}/\Delta\rho_{min}$ (e·Å^{-3})	0.38/-0.39	0.23/-0.28

Table S2. X-ray experimental data and refinement for for $(L^{dmp})_2Zn$ and $(L^{dmp})_2Mg$.

Atoms	$(L^{dmp})_2Zn$	Literature reference [S1-5]	Atoms	$(L^{dmp})_2Mg$	Literature reference [S6-8]
**Distances (Å)**					
Zn1-O1	1.8918(14)	1.926(4) – 1.897(1)	Mg1-O1	1.879(2)	1.900(3) – 1.868(3)
Zn1-N1	2.1406(17)	2.113(2) – 2.075(1)	Mg1-N1	2.205(3)	2.193(3) – 2.125(3)
**Angles (Å)**					
O1-Zn1-O1^i	119.47(9)	133.15(7) – 105.64(16)	O1-Mg1-O1^i	124.72(14)	133.19 – 125.8(1)
N1-Zn1-N1^i	120.45(9)	141.12(11) – 118.98	N1-Mg1-N1^i	119.92(15)	142.09 – 119.4(1)
O1-Zn1-N1	94.54(6)	100.3(2) – 96.83	O1-Mg1-N1	91.97(10)	96.1(1) – 92.7(1)
O1-Zn1-N1^i	114.93(7)	114.0(2) – 99.13	O1-Mg1-N1^I	115.48(10)	111.7(1) – 99.80

^{i} 1/2-X,+Y,1-Z

Table S3. Selected bond distances (Å) and angles (°) for $(L^{dmp})_2Zn$ and $(L^{dmp})_2Mg$.

Supplementary literature:

S1. Ejfler, J.; Szafert, S.; Mierzwicki, K.; Jerzykiewicz, L. B.; Sobota, P. Homo- and heteroleptic zinc aminophenolates as initiators for lactide polymerization.. *Dalton Trans.* **2008**, *46*, 6556–6562.

S2. Jędrzkiewicz, D.; Adamus, G.; Kwiecień, M.; John, Ł.; Ejfler, J. Lactide as the Playmaker of the ROP Game: theoretical and Experimental Investigation of Ring-opening Polymerization of Lactide Initiated by Aminonaphtholate Zinc complexes, *Inorg. Chem.* **2017**, *56*, 1349–1365.

S3. Farwell, J. D.; Hitchcock, P. B.; Lappert, M. F.; Luinstra, G. A.; Protchenko, A. V.; Wei, X.-H. Synthesis and structures of some sterically hindered zinc complexes containing 6-membered ZnNCCCN and ZnOCCCN rings *J. Organomet. Chem.* **2008**, *693*, 1861–1869.

S4. Ikpo, N.; Saunders, L. N.; Walsh, J. L.; Smith, J. M. B.; Dawe, L. N.; Kerton, F. M. Zinc Complexes of Piperazinyl-Derived Aminephenolate Ligands: Synthesis, Characterization and Ring–Opening Polymerization Activity. *Eur. J. Inorg. Chem.*, **2011**, 5347–5359.

S5. Zheng, Z.; Zhao, G.; Fablet, R.; Bouyahyi, M.; Thomas, C. M.; Roisnel, T.; Casagrande Jr., O.; Carpentier, J.-F. Zinc and enolato-magnesium complexes based on bi-, tri- and tetradentate aminophenolate ligands. *New J. Chem.*, **2008**, *32*, 2279–2291.

S6. Grala, A.; Ejfler, J.; Jerzykiewicz, L. B.; Sobota, P. Chemoselective alcoholysis of lactide mediated by a magnesium catalyst: an efficient route to alkyl lactyllactate. *Dalton Trans*., **2011**, *40*, 4042–4044.

S7. Eifler, J.; Krauzy-Dziedzic, K.; Szafert, S.; Jerzykiewicz, L. B.; Sobota, P. Synthesis, characterization, and catalytic studies of (aryloxido)magnesium complexes..*Eur. J. Inorg. Chem.* **2010**, 3602–3609.

S8. Shere, H.; McKeown, P.; Mahon, M. F.; Jones, M. D. Making the cut: Monopyrrolidine-based complexes for the ROP of lactide. *Eur. Polym. J.* **2019**, *114*, 319–325.
